# The green chemistry of chalcones: Valuable sources of privileged core structures for drug discovery

**DOI:** 10.3389/fchem.2022.988376

**Published:** 2022-09-12

**Authors:** Ludovica Marotta, Sara Rossi, Roberta Ibba, Simone Brogi, Vincenzo Calderone, Stefania Butini, Giuseppe Campiani, Sandra Gemma

**Affiliations:** ^1^ Department of Biotechnology, Chemistry and Pharmacy, University of Siena, Siena, Italy; ^2^ Department of Pharmacy, University of Pisa, Pisa, Italy

**Keywords:** chalcones, green chemistry, pharmaceuticals, drug discovery, green synthetic techniques, privileged structures, artificial intelligence

## Abstract

The sustainable use of resources is essential in all production areas, including pharmaceuticals. However, the aspect of sustainability needs to be taken into consideration not only in the production phase, but during the whole medicinal chemistry drug discovery trajectory. The continuous progress in the fields of green chemistry and the use of artificial intelligence are contributing to the speed and effectiveness of a more sustainable drug discovery pipeline. In this light, here we review the most recent sustainable and green synthetic approaches used for the preparation and derivatization of chalcones, an important class of privileged structures and building blocks used for the preparation of new biologically active compounds with a broad spectrum of potential therapeutic applications. The literature here reported has been retrieved from the SciFinder database using the term “chalcone” as a keyword and filtering the results applying the concept: “green chemistry”, and from the Reaxys database using the keywords “chalcone” and “green”. For both databases the time-frame was 2017–2022. References were manually selected based on relevance.

## 1 Introduction

Since many years, the pharma industry has realized that the production of medicines and pharmaceutical products needs to be accomplished by taking into account the sustainability of the manufacturing pipeline. This requisite has prompted the investigation and design of greener approaches for the production of active pharmaceutical ingredients (APIs). In particular, the “12 principles of green chemistry” should be applied not only during the manufacturing of APIs and related products, but also in all phases of the drug discovery and development workflow, starting from the early investigation of the chemical scaffolds suitable for the synthesis through green and sustainable approaches.

**FIGURE 1:**
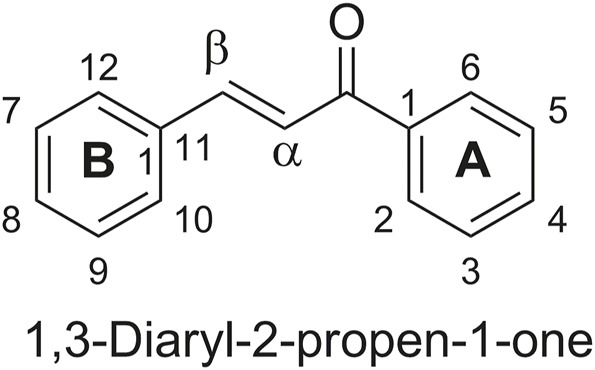
General structure of chalcone.

The use of privileged structures, defined as molecular scaffolds endowed with drug-like properties and characterized by versatile binding modes, is a design strategy massively and successfully exploited at the lead optimization stages of the drug discovery pipeline. Among the plethora of privileged structures identified so far, the chalcone framework, defined as a α,β-unsaturated carbonyl moiety substituted by two aromatic rings at both ends (general structure in Figure 1) is particularly interesting due to its widespread diffusion in the plant kingdom, food and nutraceuticals coupled to its interesting biological properties. The pharmacological profile of chalcones (Salehi et al., 2021) is wide and interesting and they are potentially useful for the development of therapeutic agents against cancer (Ouyang et al., 2021), infectious diseases (Mahapatra et al., 2015; Duran et al., 2021; Pozzetti et al., 2022), obesity, neurological/neurodegenerative disorders (Cho et al., 2011; Thapa et al., 2021), inflammatory-related syndromes (Mahapatra et al., 2017), allergic illnesses (Yamamoto et al., 2004; Iwamura et al., 2010) etc. In addition, antioxidant effects of chalcones have been demonstrated (Janković et al., 2020) conferring to them the status of privileged scaffolds. In fact, chalcone-based molecules such as metochalcone and sofalcone are used in clinical practice as choleretic drugs and anti-ulcer agents, respectively, also establishing gastroprotection in patients suffering from *Helicobacter pylori* (Higuchi et al., 2010; Gomes et al., 2017c).

Moreover, the chalcone structure represents a versatile starting material suitable for further derivatization and conversion into structurally diverse molecular entities, such as pyrrole and pyrazoles, pyridines, pyrimidines, as well as bicyclic derivatives such as indoles, quinolines and coumarins, all characterized by drug-like properties. In this scenario, green synthetic approaches for the preparation and derivatization of natural and synthetic chalcones could represent the starting point for greener and more sustainable drug discovery pipelines.

A number of reviews have focused on synthetic methodologies for the preparation and derivatization of chalcones using green approaches, often compared to standard ones, or focused on their most relevant biological activities (Rosa et al., 2017; Taresh, 2022; Wilhelm et al., 2022). Herein, we review the most recent literature (2017-to date) describing green approaches for the synthesis and derivatization of chalcone with a focus on methodologies aimed at optimizing solvent and energy consumption. The application of important green processes such as one-pot syntheses, click-chemistry, biocatalysis, and green enabling technologies such as flow chemistry and photochemistry will also be discussed in the field of chalcone synthesis and derivatization. Finally, artificial intelligence (AI) as a powerful tool for the prioritization of compound libraries will also be briefly outlined.

## 2 Green solvents for the synthesis and derivatization of chalcones

The first principle of green chemistry is to prevent waste, the fifth is the reduction of auxiliary substances as solvents and the decrease in their toxicity. However, as often reported, the greenest option for solvent is to avoid solvent. Though it is not always an easy task in the managing of organic reactions, many researchers have investigated the use of eco-friendly solvents that owe this definition to environmental reasons, low cost, time and energy waste. Amongst the many techniques encompassing procedures or devices where solvents are not necessary, several may be applied to the formation of chalcones or their derivatives. The use of ionic liquids as the catalyst allows to carry out solvent-free reactions, increasing yield products, regioselectivity, minimizing the production of chemical wastes, decreasing reaction time and simplifying operational procedures (Mahato et al., 2017; Aegurla and Peddinti, 2018; Bahrami et al., 2019; Das et al., 2020; Karimi-Jaberi et al., 2020; Karimi-Jaberi et al., 2020). Solvent-free conditions can be applied to microwave-assisted synthesis of chalcones, speeding up the reaction time. A common association is between solvent-free reactions and mechanochemical techniques, which include grinding and high-speed ball milling. These techniques are frequently used in the synthesis of chalcones and chalcone derivatives in academic research as well as in industrial applications allowing to reduce the consumption of high cost catalysts and shortening the reaction times (Praveena et al., 2019; Zangade and Patil, 2020).

### 2.1 Mechanochemistry and solvent-free synthesis

In the recent years, mechanochemistry has combined the principles of chemistry and mechanical engineering, assuming that to get a chemical transformation, energy is needed. This goal can be achieved either by using simple tools such as mortar and pestle or by more costly instruments as a ball mill (Gomes et al., 2020). The former is an easy-to-find tool but significant variations may occur depending on the operator; the latter is an automatic instrument, which leads to good reproducibility thanks to the automatic control of many parameters such as milling frequency, material and size of the milling balls and jars, the number of balls and the type of rotation (Gomes et al., 2020). Many research groups are investigating this new technology for implementing new devices which are based on the simple principle of applying a mechanical force during the reaction. Examples from recent literature are reported below where chalcone formation and chalcone derivatization reactions have been performed using the principle of mechanical chemistry.

**FIGURE 2:**
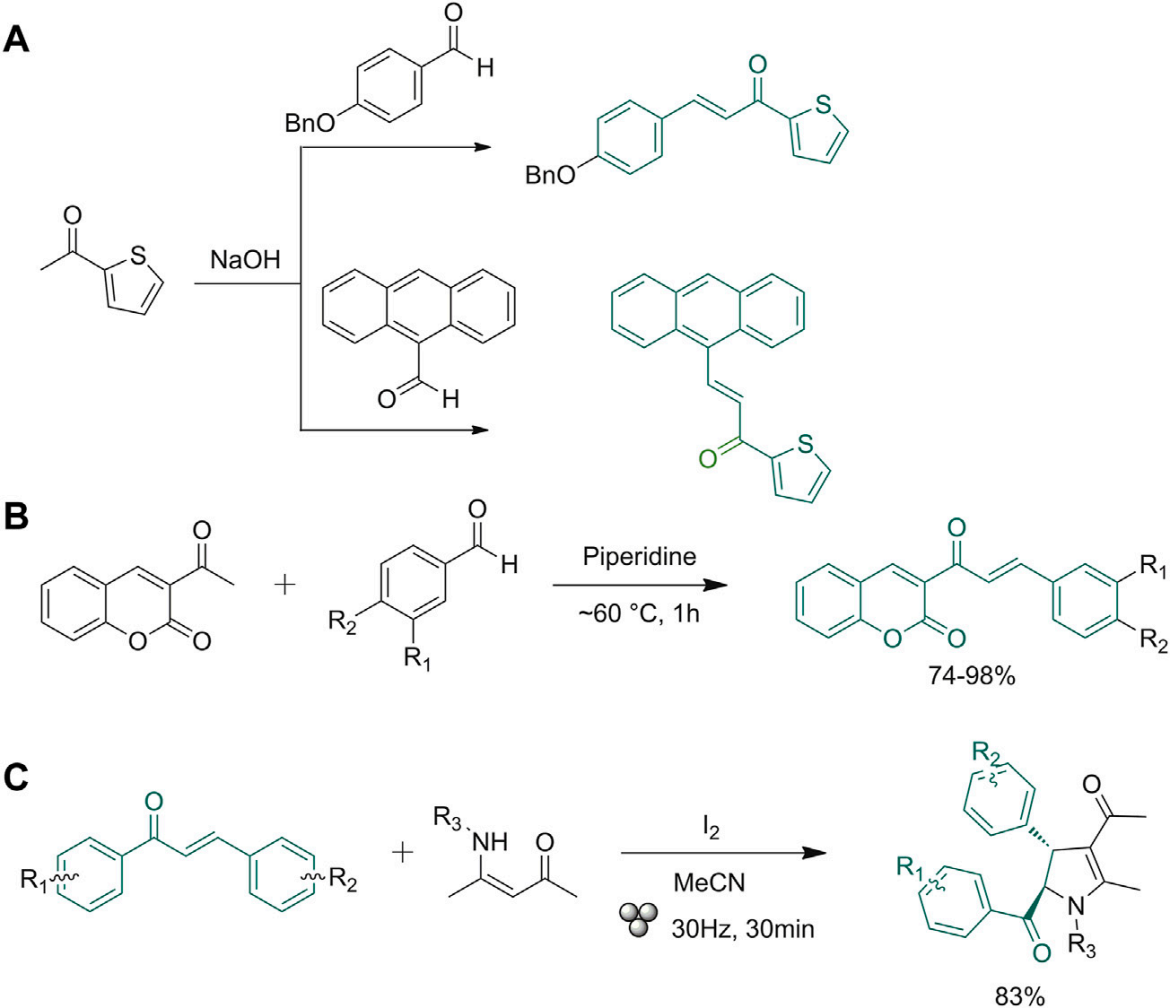
**(A)** Synthesis of anthracene- and 4-benzyloxy-based chalcones; **(B)** Claisen-Schmidt condensation between 3-acetyl-2*H*-chromen-2-one mono- and disubstituted benzaldehydes; **(C)** synthesis of polysubstituted 2,3-dihydropyrroles (relative stereochemistry is shown).

Praveena *et al.* described two examples of reactions carried out through the use of pestle and mortar (Praveena et al., 2019). As exemplified in Figure 2A, 4-(benzyloxy)benzaldehyde or 9-anthracenecarbaldehyde were mixed with 1-(thiophen-2-yl)ethanone and NaOH and then grinded with mortar and pestle for 10 min. The formed solid chalcones were filtered off and re-crystallized from ethanol (Praveena et al., 2019). Gomes *et al.* used a laboratory device consisting of a single-screw drill (SSD) placed on top of a steel cylinder with a central hole, where the drill screw penetrates once operated. A friction force is generated using this technique, resulting in an intense shear stress able to reduce the powders down to 1 µm in 1 min and create a properly mixed reaction, subjecting the system to high pressure. This technique has been tested on the classical Claisen−Schmidt condensation reaction using various aldehydes and methyl ketones as starting materials (Gomes et al., 2020). The same experiment was carried out using the ball milling technique. The SSD tool, which uses the shear force instead of shock pressure, allows a higher availability of the molecular surface, increasing the reaction yields (Gomes et al., 2020). The SSD method provided chalcones in high yields regardless of the physical state (solid or liquid) or the presence of functional groups (electron-donating, electron-withdrawing) on the starting material scaffolds. The aim of the research group of Cuellar and co-workers was to synthesize active pharmacological coumaro-chalcone compounds by a sustainable route (Figure 2B). They applied a classic Claisen-Schmidt condensation without solvent, obtaining high yields (75.2–99.4%) with the selective formation of the trans isomer in the chalconoid system (Cuellar et al., 2022). Exploiting the mechanochemistry technique, another research group performed reactions using nitrones as a replacement for aldehydes. In the experiments, they verified that the reaction yield with nitrone derivatives and acetophenone does not change as compared to the use of aldehydes. They also tested the addition of small amounts of xylene, a high boiling non-polar solvent, for facilitating the grinding mechanism (Antony et al., 2021).

Being able to facilitate reaction conditions in the presence of a small amount of solvent (or any), ball milling devices have several applications in green organic synthetic approaches. Weng *et al.* (Weng et al., 2021) exploited this technique for derivatizing the scaffold of chalcones. Starting from a simple condensation reaction, they developed a protocol for preparing a library of polysubstituted 2,3-dihydropyrrole derivatives (Figure 2C). This reaction was performed via iodine-promoted cyclization with the use of additives such as liquid-assisted grinding solvents (LAGs). Different solvents were tested, with different starting material equivalents and different time rates, until the reaction was optimized, and high yields were obtained. The optimization of the above-described protocol allowed to create a consistent library of 2,3-dihydropyrroles, and to study the effects of substitution patterns on reactivity.

### 2.2 Micellar chemistry

Water may be considered as the greenest solvent due to several inherent properties (i.e., water is neither toxic, flammable or carcinogenic, it is readily available and inexpensive). Unfortunately, the use of water as a solvent for organic chemistry reactions presents many limitations, the most important being the poor solubility of most reagents. To overcome this problem, surfactants are used tot increase the miscibility among hydrophobic substances and water. Due to their amphiphilic nature, in the presence of a large amount of water, surfactants form micelles, defined as spherical supramolecules with a colloidal size (Wei and Cue, 2018). The “supramolecule” term indicates that cohesion forces are not covalent and even if the micelles appear as a homogeneous phase, they consist of a micro-heterogeneous two-phase system. Tensides are used in many industrial applications, in chemical synthesis they are used as micellar catalysts able to accelerate or inhibit a reaction process (La Sorella et al., 2015).

**FIGURE 3:**
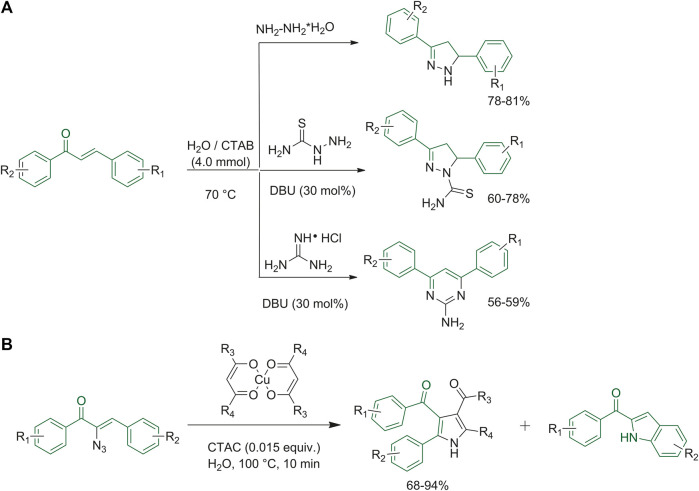
**(A)** Synthesis of 4,5-dihydro-1*H*-pyrazoles and 4,6-diphenyl-pyrimidin-2-amine by micellar reactions; **(B)** synthesis of pyrrole derivatives in a micellar medium.

Starting from appropriately substituted chalcones, Mishra *et al.* studied a new procedure to synthetize the 4,5-dihydro-1*H*-pyrazole and 4,6-diphenylpyrimidin-2-amine to prevent environmental pollution and reduce the extensive use of organic solvents. They chose cetyltrimethylammonium bromide (CTAB) as the cationic surfactant for the micelles formation and added 1,5-diazabiciclo(5.4.0)undec-7-ene (DBU) as the base catalyst. They screened different molar concentrations and several bases, gaining the best yields when they used the conditions reported in Figure 3A (Mishra et al., 2017). Rajaguru *et al.* aimed at synthesizing indoles by reacting α-azidochalcones in the presence of metal β-diketonates using cetyl trimethyl ammonium chloride (CTAC) in water as the micellar medium. During their experiments they could obtain substituted pyrroles, and for this reason they extended the study of reaction conditions in order to improve the yields of the pyrrole derivatives, as depicted in Figure 3B (Rajaguru et al., 2017).

Kothandapani and co-workers started with the synthesis of the non-ionic surfactant stearyl methoxyPEGglycol succinate (SMPS). This compound emerged as an interesting option due to its non-toxic nature. Non-ionic surfactants SMPS can be modified by changing the length of the hydrophilic tail to improve their aqueous miscibility, while the substitution of different alcohols to 1-octadecanol gave different degrees of lipophilia (Kothandapani et al., 2017). To test the efficacy of the surfactant that they synthesized, the research group applied the reduction reaction of nitrochalcone derivatives to the classical approach employing palladium/diphenyl sulfide complex catalyst, which is expensive and toxic. Consequently, Kothandapani *et al.* performed the reduction by SMPS surfactant in aqueous medium with zinc and ammonium chloride. The efficiency of SMPS was verified by carrying out the reaction under the same conditions without the surfactants (Kothandapani et al., 2017).

### 2.3 Ionic-liquid synthesis

In chemical processes, organic solvents are used in enormous amounts because they are needed during reaction, separation, and formulation and, depending on an appropriate selection, they can lead to good yields and bring a quality product. The use of less toxic solvents would have an important impact on the sustainability of the API manufacturing process. In the last few years, ionic liquids have emerged as a green alternative to common organic solvents. By definition, ionic liquids are salts with a low melting point and are characterized by physicochemical properties such as negligible vapor pressure, high thermal stability, and wide liquid range; moreover, these features can be modulated by combining two ILs or by modifying the length and flexibility of the organic cation. This accounts for their high adaptability to different specific applications. On the other hand, the lack of data about endpoints and toxicity profiles, their tendency to combustion, high cost and low potential biodegradability led to the conclusion that ILs still need to be studied and optimized (Wei and Cue, 2018).

**FIGURE 4:**
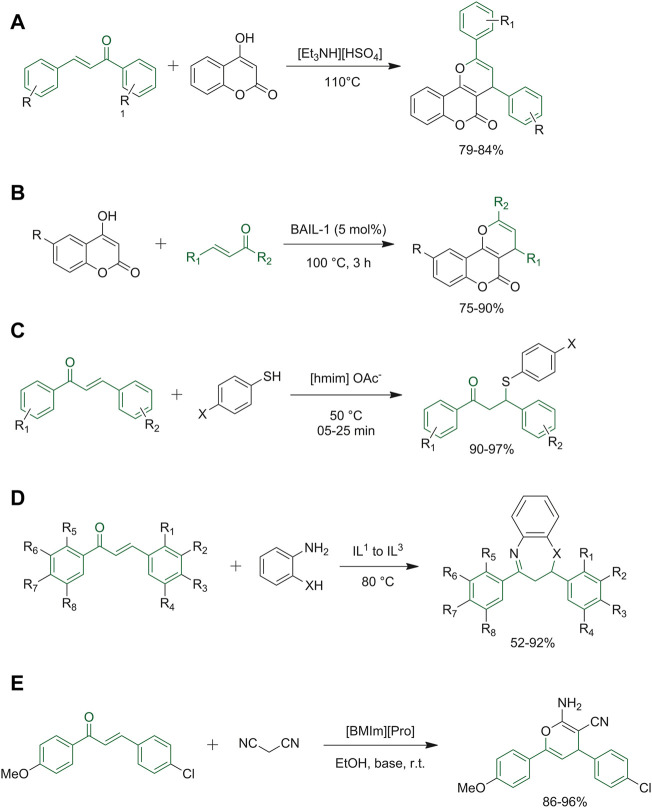
**(A)** Synthesis of pyrano [3,2-*c*]coumarins; **(B)** synthesis of pyrano [3,2-*c*]coumarin using BAIL-1 as liquid ionic; **(C)** synthetic route for 1,3-diphenyl-3-(phenyl thio) propan-1-one derivatives; **(D)** Michael addition followed by cyclo-condensation to give 1,5-benzodiazepines and 1,5- benzothiazepines; **(E)** formation of the 2-amino-3-cyano-4*H-*pyrano.

Triethylammonium hydrogen sulfate is considered an environmentally safe and cheap ionic liquid catalyst, which is easily prepared by an acid-base neutralization reaction. This catalyst has been successfully employed in the synthesis of pyrano[3,2-*c*]coumarins by the reaction of 4-hydroxycoumarin with differently substituted chalcones (Figure 4A) (Karimi-Jaberi et al., 2020). Pyrano[3,2-*c*]coumarines were synthesized by Mahato *et al.* using Brønsted acidic ionic liquids (BAILs) (Figure 4B). Firstly, the authors applied solvent-free conditions coupled to heating at high temperatures, obtaining satisfactory yields. Then they also investigated different *ad hoc* synthesized ILs, and the best result was obtained by BAIL-1, a recyclable IL that can be easily purified with water being the only byproduct (Mahato et al., 2017). 1,4-Addition of mercaptans to chalcones was obtained under a variety of reaction conditions, characterized by heating at high temperatures, the need of expensive catalysts, and long reaction times. Shreyas S. Mahurkar and co-workers developed a green method for the synthesis of 1,3-diphenyl-3-(phenylthio)propan-1-one derivatives using [hmim]OAc-ionic liquid for the thia-Michael addition reaction (Figure 4C). This ionic liquid was effective as a reaction medium and a good catalyst. The authors hypothesized that the dissociation constant of thiophenol, which is higher in ionic liquids than in organic solvents, could be responsible for the improved reaction outcomes (Mahurkar et al., 2019). Sakirolla *et al.* employed imidazolium salt ionic liquids to synthetize 1,5-benzothiazepine starting from α,β-unsaturated carbonyl systems typified by chalcones (Figure 4D). These ILs have proven to be environmentally friendly since they can be recycled for three cycles, IL-C is the most efficient reaction medium among the others because it allowed to reduce the time reaction and increase the final yield (Sakirolla et al., 2018). Recently, the research group of Dhadda, has applied together with the use of ionic liquids with the technique of sonication and managed to create a scheme for different synthetic heterocyclic compounds, including 2-amino-3,4-pyrimidine, pyrazole, oxazole, and pyridine derivatives. The basic ionic liquid [DBUH]OAc has proven to be the best IL since it leads to obtaining good yield of the desired result and it can also be reused for up to five catalytic cycles (Dhadda et al., 2022).

Besides being used as solvents, ILs have also been used purely as catalysts. Amino acidic-based ionic liquids such as *l* -proline nitrate and [BMIm]-proline have been used as green and biodegradable catalysts in conjugate addition reactions with chalcones (Bahekar et al., 2017). In this case, the use of a *l* -proline nitrate avoids the need of long reaction times and high-temperature, which are typical reaction conditions required for thia-Michael reactions; at the same time, high yields and environmental sustainability were attained [BMIm]-proline proved to be an efficient ionic liquid catalyst allowing the synthesis of various 2-amino-4*H*-chromene-3-carbonitrile derivatives in high yields. The catalyst can be recycled up to five times still giving good yields, though the recovery process can be time-consuming (Figure 4E) (Hajipour and Khorsandi, 2017).

## 3 Synthesis of chalcones using green sources of energy

In the last 30 years, ultrasound technique has encountered increasing interest in the field of drug synthesis as an advantageous methodology for more sustainable manufacturing approaches. Indeed, the mechanism of the ultrasound system, also called sonochemistry, consists of the generation of a high amount of energy released by the collapse of microbubbles. Microbubbles are formed by piezoelectrical materials subjected to electrical potential, these materials convert electrical energy to mechanical vibration energy letting it spread through the liquid medium to form microbubbles (Wei and Cue, 2018; Draye et al., 2020). These microbubbles absorb energy from the waves generated by piezoelectrical materials and grow until they implode. This localized explosion with extreme conditions of temperature and pressure allows the reaction to take place (Draye et al., 2020).

In the last decades, the microwave technique has proven to be effective in increasing reaction yields and reducing both reaction times and the formation of side products. In contrast to conventional heating, microwave irradiation warms up the solution homogeneously and rapidly. Microwave frequencies work as electric fields that generate heat when they encounter materials with dielectric properties. Indeed, the selectivity of the reaction can be enhanced by the knowledge that polar molecules are more affected by microwave irradiation than apolar ones. This technique falls into the sixth principle of green chemistry since the energy efficiency is increased. Despite this, the microwave is still considered ambiguous as a green process because only 65% of electrical energy is converted into electromagnetic radiation, and the radiation heat can be slow when the mixture reaction is apolar. Nonetheless, microwave chemistry is widely used for solvent-free reactions or with the support of other devices such as flow chemistry devices (De La Hoz et al., 2016).

## 3.1 Ultrasound chemistry

**FIGURE 5:**
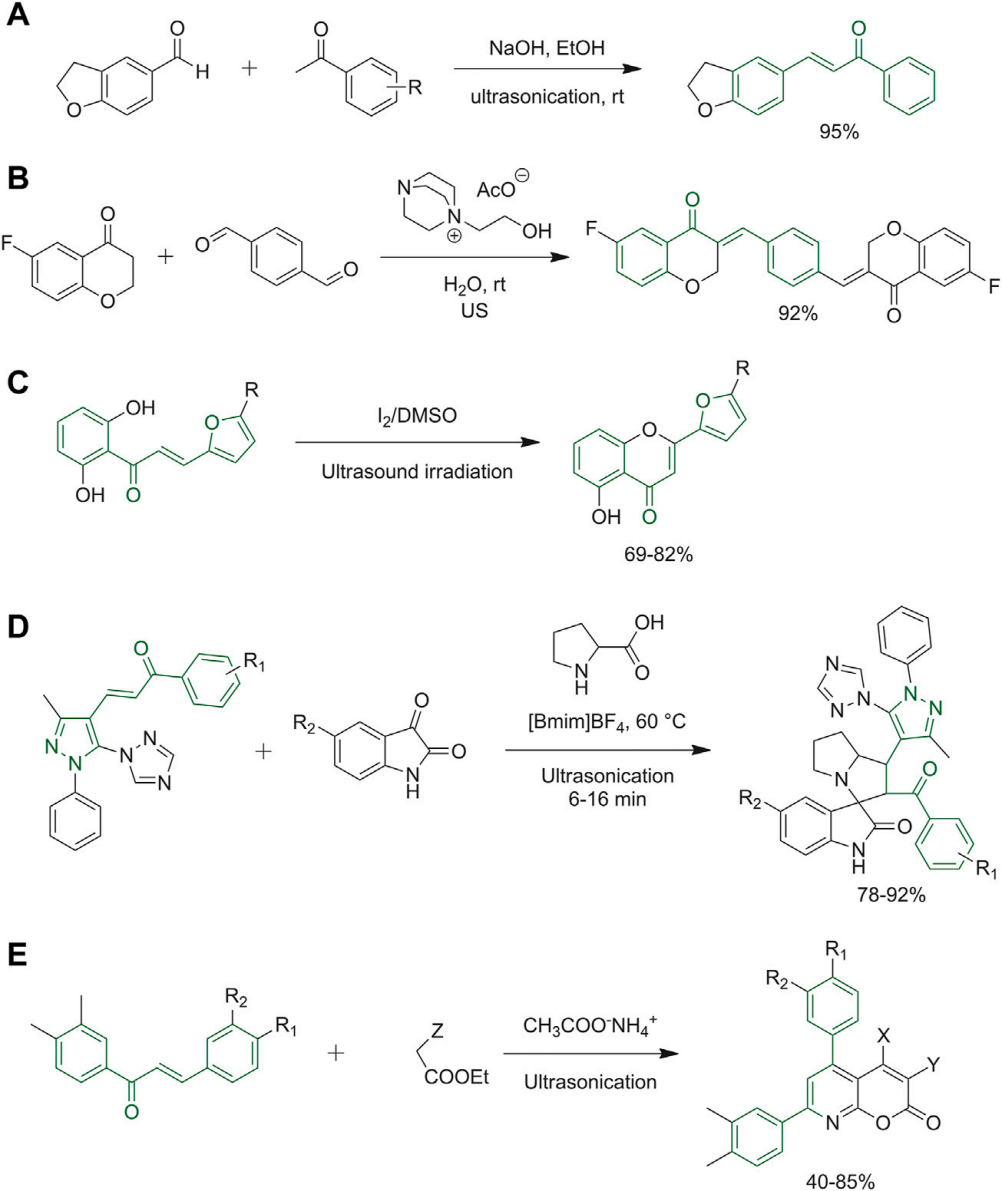
**(A)** Synthesis of **(E)**-3-(2,3-dihydrobenzofuran-5-yl)-1-(aryl)prop-2-en-1-one derivatives; **(B)** 6-fluorochroman-4-one and terephthalaldehyde are used for a Claisen–Schmidt condensation to obtain bischalcone by ultrasound method and DABCO-based IL; **(C)** the last synthetic step to obtain 2-(5-substituted-furan-2-yl)-4*H*-chromen-4-ones; **(D)** final step of ultrasonication-ionic liquid synergy to obtain 1,2,4-triazol-1-yl-pyrazole based spirooxindolopyrrolizidine derivatives; **(E)** ultrasonication reaction between chalcone derivative and ethyl-substituted acetate obtaining pyrano [2,3-*b*]pyridine.

Adole and co-workers reported the first synthetic route to obtain (*E*)-3-(2,3-dihydrobenzofuran-5-yl)-1-(aryl)prop-2-en-1-one derivatives under ultrasound irradiation. Their goal was to simplify the work-up procedures. The best identified reaction conditions consisted in reacting 2,3-dihydrobenzofuran-5-carbaldehyde with substituted acetophenones in ethanol, in the presence of sodium hydroxide, under ultrasound irradiation at room temperature (Figure 5A) (Adole et al., 2020). Sharma and co-workers developed an approach to get coumarin hybrids with other molecules such as maleimide, α-lipoic acid and resveratrol, thus combining two biologically active structures and also synthesisized a set of chalcones presenting coumarin heterocycle as the ring B (Sharma et al., 2018). Arafa et *al* had the goal of finely-tune a new green protocol to synthesize bis-chalcones through a Claisen–Schmidt condensation. Based on their knowledge of ILs as sustainable solvents and the ultrasound techniques, they optimized the reaction between 6-fluorochroman-4-one and terephthalaldehyde, which occurred with poor yields when using conventional methods, using ethanol as the solvent, at room temperature, and using sodium hydroxide as the base. A slight improvement was attained using the ultrasound method. Subsequently, both the solvent and the base catalysts were changed, and the best yields were obtained with DABCO-based IL [DABCO-EtOH][AcO] coupled to ultrasonication, as reported in Figure 5B. By using these optimized reaction conditions, several bis-chalcone derivatives have been obtained (Arafa, 2018). Kakade *et al.* synthesized 2-(5-substituted-furan-2-yl)-4*H*-chromen-4-ones from furan-substituted chalcones, in dimethyl sulfoxide in the presence of a catalytic amount of iodine under ultrasound irradiation at room temperature conditions (Kakade and Vedpathak, 2020). The products depicted in Figure 5C were obtained within a few minutes in good yields. Pogaku *et al.* developed a green procedure joining together the IL 1-butyl-3-methlimidazoliumtetrafloroborate ([Bmim]BF4) and ultrasonication. Figure 5D represents the one-pot reaction among a chalcone intermediate, isatin and *l* -proline to obtain the final product 1,2,4-triazol-1-yl-pyrazole-based spirooxindolopyrrolizidine derivatives. The identified optimal conditions exploited [Bmim]BF_4_ with ultrasonication at 60°C. The use of classical solvents such as methanol at room temperature or under reflux resulted in no reaction or a very low yield of the spirocyclic heterocycles (Pogaku et al., 2019). The reaction reported in Figure 5E represents the synthesis of a series of pyrano [2,3-*b*]pyridines elaborated by Rizk group. This procedure allowed to achieve high yields under mild conditions, starting from chalcone derivatives and reacting them with ethyl-substituted acetate under ultrasonic radiation. The ethyl-substituted acetates used were diethylmalonate, ethylacetoacetate or ethylcyanoacetate. The final pyrano [2,3-*b*]pyridines obtained differ in the substitution of substituents X and Y depending on the nature of the ethyl-substituted acetate used and the number of reactants such as via three- or four-component reactions. The same reaction was performed by grindstone technology providing equally high yields (Rizk et al., 2017).

Oxyprenylated chalcones of natural origin, such as cordoin and 4-hydroxycordoin, have interesting biological properties. Villena *et al.* have recently investigated the synthesis of oxyprenylated derivatives through alkylation of 2′,4′-dihydroxychalcone with different alkyl bromides as the first step of the synthesis in the presence of a slightly basic medium and under ultrasound conditions. The reaction mixture was irradiated in a water bath by an ultrasonic cleaner. The ultrasonic system allowed to reduce reaction time, improving the overall alkylation yields (Villena et al., 2021). Several aryl/indolyl substituted 4,5-dihydro-1*H*-pyrazole derivatives were synthesized by Kannan and co-workers. Chalcones exposed to aryl hydrazides undergo a Michael addition-cyclization reaction, forming the corresponding pyrazole scaffolds. This reaction was carried out both by conventional method and ultrasonic irradiation, the latter allowed to decrease the reaction time with a comparable yield (Kannan et al., 2019). Thanks to the success of the previous reaction, Kannan and co-workers extended the synthetic process to indole-based chalcones by reacting them with different carbohydrazides following the same ultrasonication conditions reported above (Kannan et al., 2019). Finally, a one-pot cyclo condensation reaction was performed between chalcone derivatives and *o*-aminothiophenol by Devkate *et al.* In their approach, several solvents were tested and PEG-400 was identified as the best choice providing a shorter reaction time and straightforward separation procedures for the target 1,5-benzothiazepines (Devkate et al., 2018).

### 3.2 Microwave chemistry

Microwave irradiation is widely used for the synthesis of chalcones and their derivatives. Chalcone synthesis is commonly realized through conventional Claisen-Schmidt condensation at high temperatures. With conventional heating, the reaction times can rise to 24 h depending on the general structure of the starting materials; moreover, the reaction yields are often not satisfactory. For these reasons, new and green synthetic strategies using microwave irradiation are more and more popular among researchers.

Prabhakar and co-workers developed a green protocol for the synthesis of 9-anthracenyl chalcone derivatives, which were tested as anti-bacterial compounds against *Staphylococcus aureus* and *Bacillus subtilis*. The reaction was carried out under solvent-free conditions, using KOH as the catalyst and under microwave irradiation, giving excellent yields in 5 min (Prabhakar et al., 2017). This protocol can be applied to scaffolds bearing different functional groups as reported by Sahoo and co-workers (Sahoo et al., 2019; Tupare and Pawar, 2020). Comparative studies to prove the effectiveness of microwave-assisted synthesis over conventional methods for chalcone synthesis showed that the conventional synthesis of chalcones starting from *o*-hydroxyacetophenones requires longer reaction times, higher solvent amounts and provides lower yields when compared to microwave-assisted protocols (Shntaif, 2016; Sahoo et al., 2017; Rahim et al., 2018).

**FIGURE 6:**
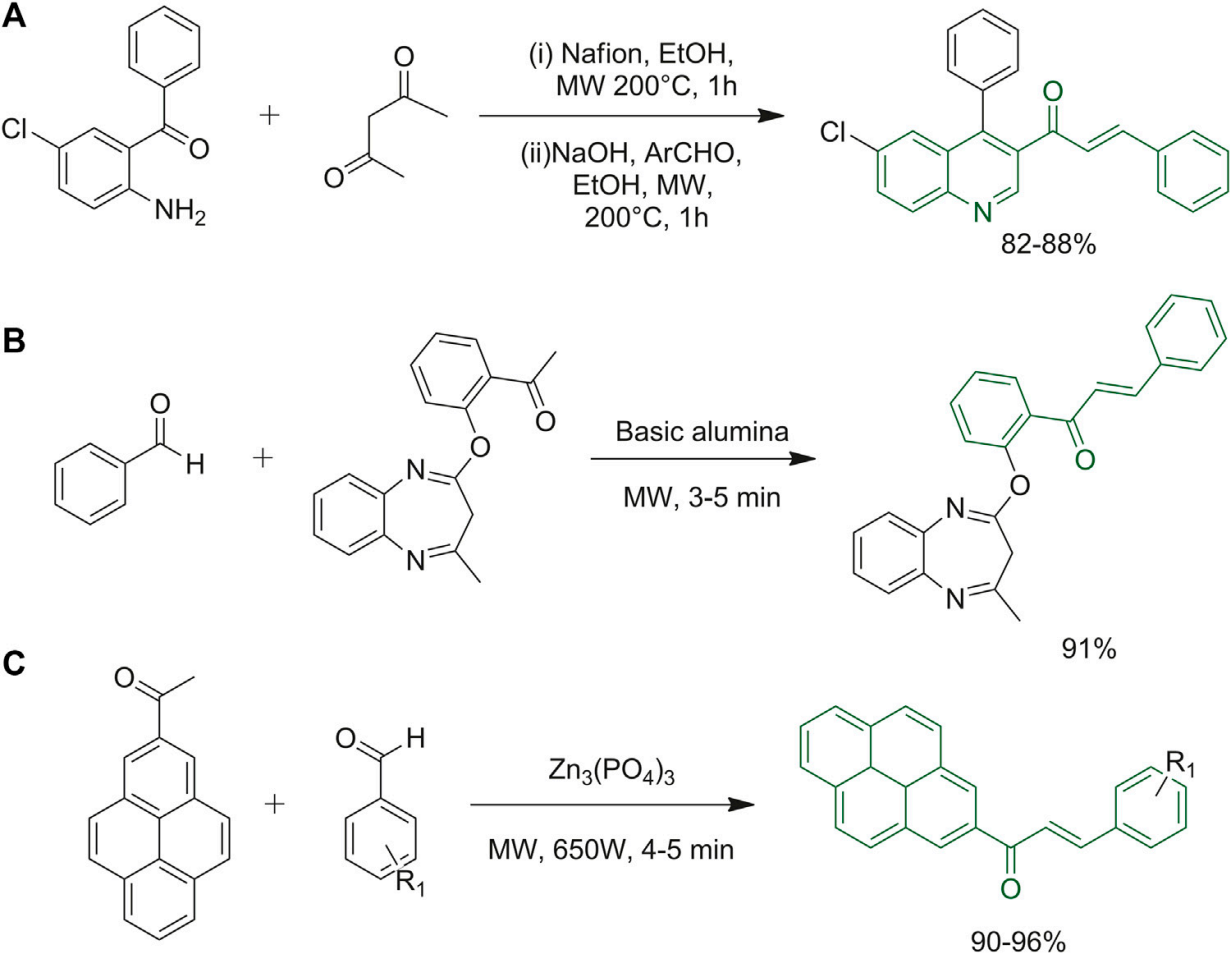
**(A)** One-pot synthesis of quinolinyl chalcones; **(B)** synthesis of 3*H-*benzo [*b*][1–4]diazepine derivatives; **(C)** synthesis of styryl 2-pyrenylketones using zinc phosphate as the coupling reagent.

Wang *et al* applied an original one-pot synthesis, made of two steps, to obtain quinolinyl chalcones (Figure 6A). They used Nafion NR50 as the catalyst in EtOH; it is a reusable synthetic polymer with ionic properties that acts as an acidic catalyst enhancing the yield rate and allowing a more sustainable synthesis of this important scaffold (Chan et al., 2020). Chalcones were combined with the well-known 1,5-benzodiazepine moiety to evaluate the biological activity of these complex poly-functionalized heterocyclic scaffolds (Figure 6B). This one-pot microwave-assisted synthetic strategy avoided the use of toxic solvents and provided good yields and avoided the need of purification by column chromatography. The desired starting materials and basic alumina were grinded in a mortar with the minimum quantity of solvent, and then the reaction mixture was irradiated with microwaves until reaction completion (Balyan et al., 2020). Muthuvel and co-workers used microwave irradiation to synthesize styryl-2-pyrenylketones using zinc phosphate (Figure 6C). The solvent-free procedure was carried out with various benzaldehydes, the ones with electron-donating groups gave better yields than the ones with electron-withdrawing functional groups. The synthetic route was tested up to 6 times to verify the reusability of the catalyst and the efficiency was not affected until the fifth reuse (Muthuvel et al., 2021).

**FIGURE 7:**
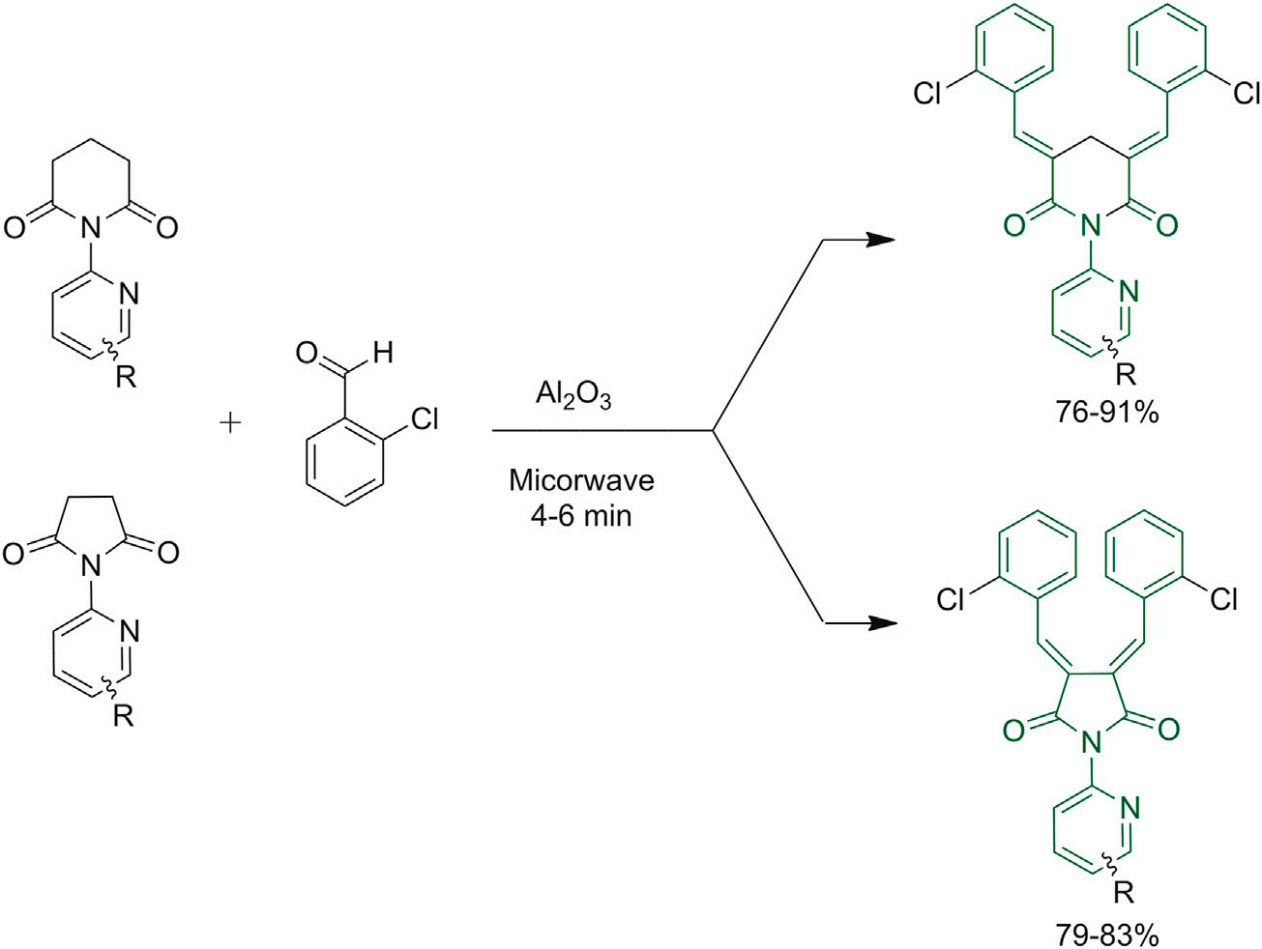
Routes to synthesize bischalcone derivatives.

A solvent-free protocol was also tested by Chaudhari *et al.* as reported in Figure 7. The routes to obtain the bis-chalcones started with the reaction of an aromatic aldehyde with *N*-phenylpyrrolidine-2,5-dione and *N*-phenyl piperidine-2,6-dione (Chaudhari and Rajput, 2018).

Li *et al.* studied the use of eco-friendly catalysts in the classical Claisen-Schmidt condensation synthesis of chalcones. Reduced graphene oxide (RGO) is widely used and studied for many industrial applications due to its chemical-physical properties, likewise, ZnO nanoparticles have received a greater interest as a catalyst in chemical reactions. The mixture of ZnO nanoparticles dispersed on RGO (Zn/RGO) results in good catalytic activity. This is often used during photocatalysts or photodegradation reactions, but Li *et al.* evaluated its efficacy in the Claisen-Schmidt condensation using different aryl aldehydes and aryl ketones with a mixture of ethanol and water as sustainable solvents under microwave heating. Further research reported that the electronic and steric hindrance effects of functional groups and electron-withdrawing groups were the most effective (Li et al., 2017). The synthesis of 4-piperidiniphenylenone derivatives was carried out by Ranganathan *et al.* The reaction was performed by a microwave-assisted protocol with solid Cu^2+^/Zeolite catalyst under solvent-free conditions. This approach highlighted that benzaldehydes with electron-donating substituents gave higher yields than those with electron-withdrawing substituents; the best concentration of catalyst was also assessed (Ranganathan et al., 2020).

**FIGURE 8:**
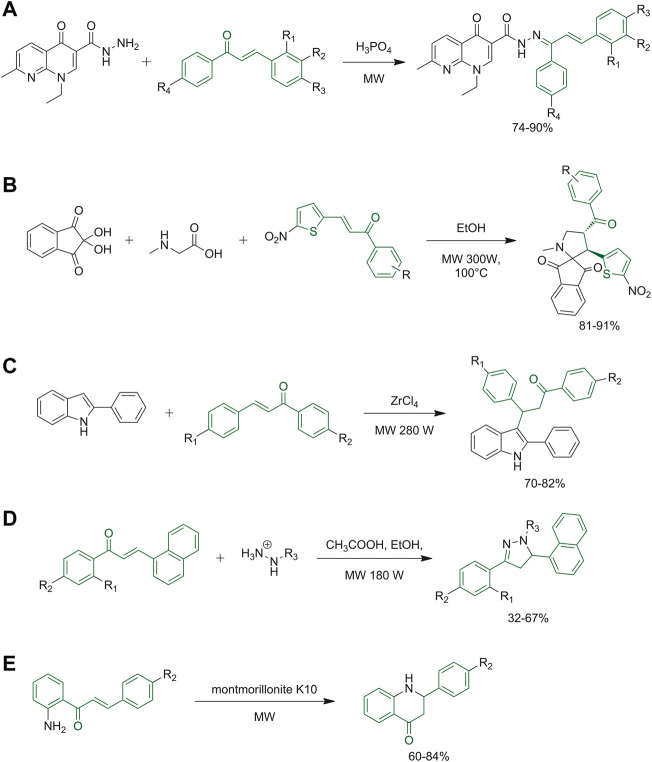
**(A)** Synthetic step of *N*′-(1,3-diphenylallylidene)-1-ethyl-7-methyl-4-oxo-1,4-dihydro-1,8-naphthyridine-3-carbohydrazide derivatives; **(B)** synthesis of novel spiropyrrolidine derivatives; **(C)** synthesis of 3-(3-oxoaryl) indole derivatives; **(D)** synthesis of pyrazoline derivatives; **(E)** the final step to synthesize 2-aryl-2,3-dihydroquinolin-4(1*H*)-ones by microwave technique.

As previously mentioned, chalcones are not only important biological scaffolds, but they are also useful synthons. The growing interest in the synthesis of hybrid molecules led Mubarak *et al.* to study a new scaffold obtained by the condensation of nalidixic acid and chalcones to obtain several *N*′-(1,3-diphenylallylidene)-1-ethyl-7-methyl-4-oxo-1,4-dihydro-1,8-naphthyridine-3-carbohydrazides. Figure 8A reports the green synthetic step used for the preparation of the product, where microwave irradiation was used to increase the yield and decrease the reaction time. To assess the sustainability of the protocol, metrics of atom economy, carbon efficiencies and reaction mass efficiency were calculated, as well as energy consumption data (Mubarak et al., 2019). Kalluraya and co-workers reported a regioselective synthesis of spiropyrrolidine derivatives in a comparative study in which microwave-assisted reactions afforded the best results in facilitating the condensation of starting materials and afterwards in the formation of the final regioselective cycloadducts (Figure 8B). The reactions were tested with and without the use of solvent, obtaining comparable results (Kalluraya et al., 2018). In the same way, a new protocol, for the synthesis of 3-(3-oxoaryl)indole derivatives, was developed through a microwave-assisted Michael addition of the appropriate chalcone derivatives using commercially available and non-toxic catalyst under solvent-free conditions (Figure 8C) (Patel et al., 2019). 1,5-Benzodiazepine derivatives were synthesized with good yields and without the need for column chromatography purification, starting from differently substituted chalcones and 2-aminoaniline dissolved in piperidine and ethanol with the aid of a domestic microwave (Shetye and Pawar, 2017). Similarly, pyrazoline derivatives were synthesized from the appropriate chalcones and hydrazine hydrate derivatives with the addition of a catalyst in ethanol under microwave irradiation (Figure 8D) (Jasril et al., 2019). Rocha and co-workers synthesized the 2′-aminochalcones derivatives using a green protocol employing microwave radiations. After that, they submitted the chalcone derivatives to a cyclization reaction to obtain 2-aryl-2,3-dihydroquinolin-4(1*H*)-ones (Figure 8E) (Rocha et al., 2019).

## 4 Tools and strategies for improving the efficiency of chalcone synthesis and derivatization

### 4.1 Click chemistry

Click chemistry has been well known since 2001, when Prof. Barry Sharpless conceived its definition. Click reactions must be modular and versatile, give high yields, be stereospecific, easy to perform and must create only harmless by-products (Shankaraiah et al., 2020). This methodology is regarded as a powerful tool for green chemistry applications due to properties such as no or few by-products, atom economy, use of catalysts (re-usable), compatibility with water and benign reaction conditions (Gupta et al., 2020). Chalcones containing alkyne-based substituents at the A or B rings were found to be compatible with the azide-alkyne Huisgen cycloaddition leading to the 1,2,3-triazole conjugates. Some examples are reported below.

**FIGURE 9:**
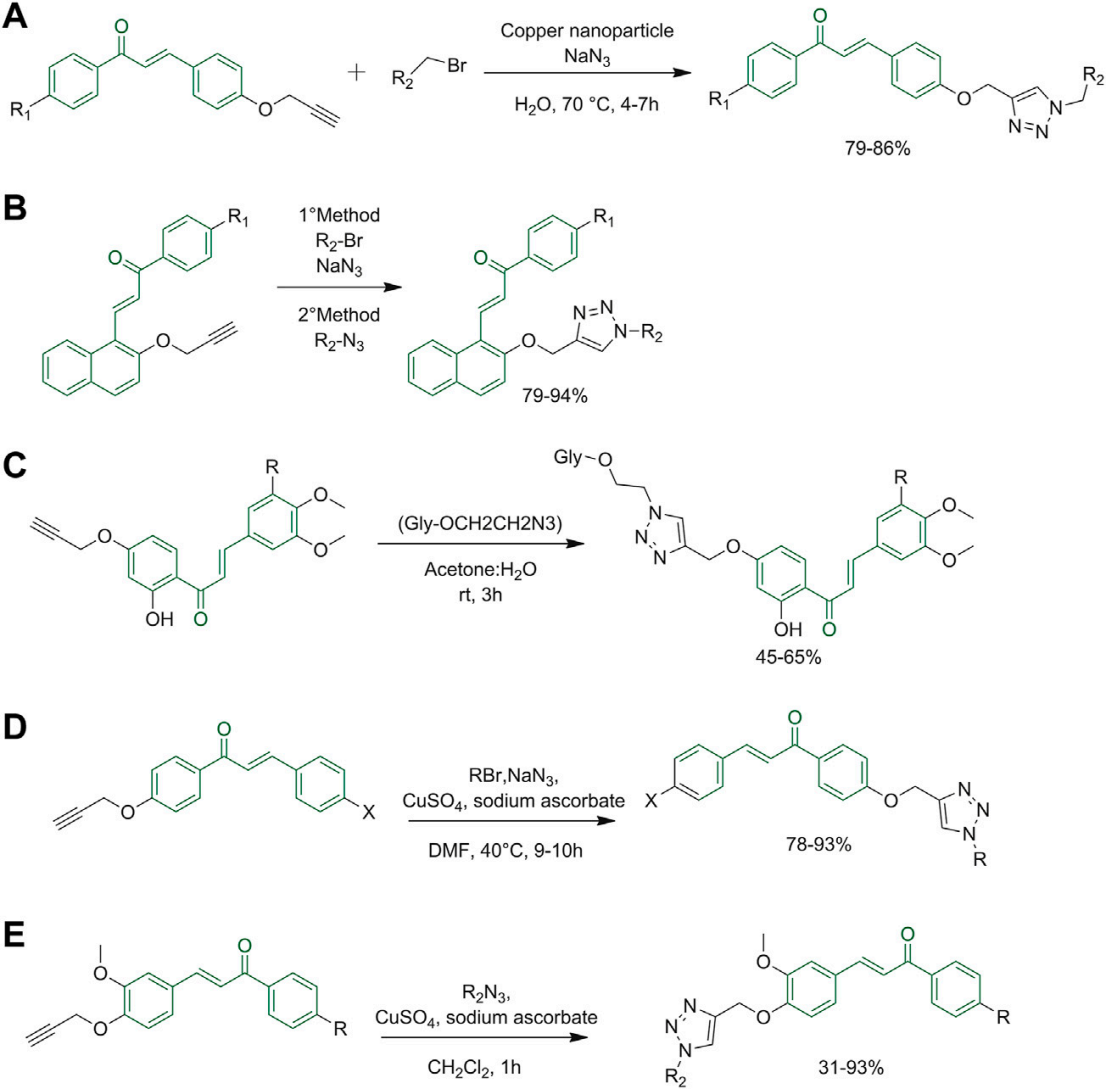
**(A)** Synthesis of chalcone derivatives starting with 4-*O*-propargylated benzaldehyde; **(B)** synthesis of 1,2,3-triazole derivatives using propargylated chalcones and azides; **(C)** click reaction copper**(I)**-catalyzed azide-alkyne cycloaddition (CuCAA) to obtain flavonoid glycosides with triazole moieties; **(D)** triazole synthesis through one-pot click reaction; **(E)** triazole synthesis through one pot click reaction catalyzed by CuSO_4_ and Na-Asc.

The research group of Yadav reacted 4-*O*-propargylated chalcone derivatives with organic azides and various benzyl bromides. Importantly, the reaction was performed in the presence of cellulose-supported copper nanoparticles (Figure 9A), which proved stable (reusable without losing any significant activity) and chemoselective for Huisgen cycloaddition, justifying their increasingly widespread use (Yadav et al., 2017). Yadav *et al.* also tested the same reaction on different scaffolds. As reported in Figure 9B, a click reaction between propargylated chalcones and azides using cellulose-supported copper nanoparticles was performed using two different reaction conditions. In the first method, chalcone conjugates were reacted with sodium azides and various benzyl bromides (R_2_-Br), while in the second method, the previously functionalized azides (R_2_-N_3_) were used. Both methods gave 1,2,3-triazoles in good yields (Yadav et al., 2018). Pereira *et al.* also reported the preparation of chalcone derivatives bearing a 1,2,3-triazole moiety. These compounds were obtained, as represented in Figure 9C, using a click reaction carried out with assisted copper(I)-catalyzed azide-alkyne cycloaddition (CuCAA). *O*-propargyl intermediates and azide sugar derivatives were the elected reagents (Pereira et al., 2021). Similarly, Om and co-workers synthesized chalcone-triazole hybrids, which showed a synergetic biological activity when compared to the two pharmacophoric moieties taken separately. The one-pot synthesis of the azide and subsequently of the triazole is carried out in DMF using an aqueous solution of sodium azide, 2-bromo-*N*-arylacetamides, and the propargylated chalcone, using copper sulfate pentahydrate and sodium ascorbate as catalysts (Figure 9D) (Sharma et al., 2022). The click reaction can be carried out under ultrasonication to enhance the reaction rate as reported by Kaur, the reaction was performed with variably substituted aromatic azides and terminal alkynes (Figure 9E) (Kaur et al., 2022).

### 4.2 One-pot synthesis

One-pot reactions consist of a sequence of several synthetic transformations carried out in a single pot avoiding purification steps of intermediate compounds. A one-pot procedure can form several chemical bonds in one-row synthesis also allowing the formation of very complex molecules. It can be considered a green approach since the use of solvents and chemical waste is minimal, harsh reaction conditions are avoided and the simplification of the synthetic routes is maximized (Sydnes, 2014).

**FIGURE 10:**
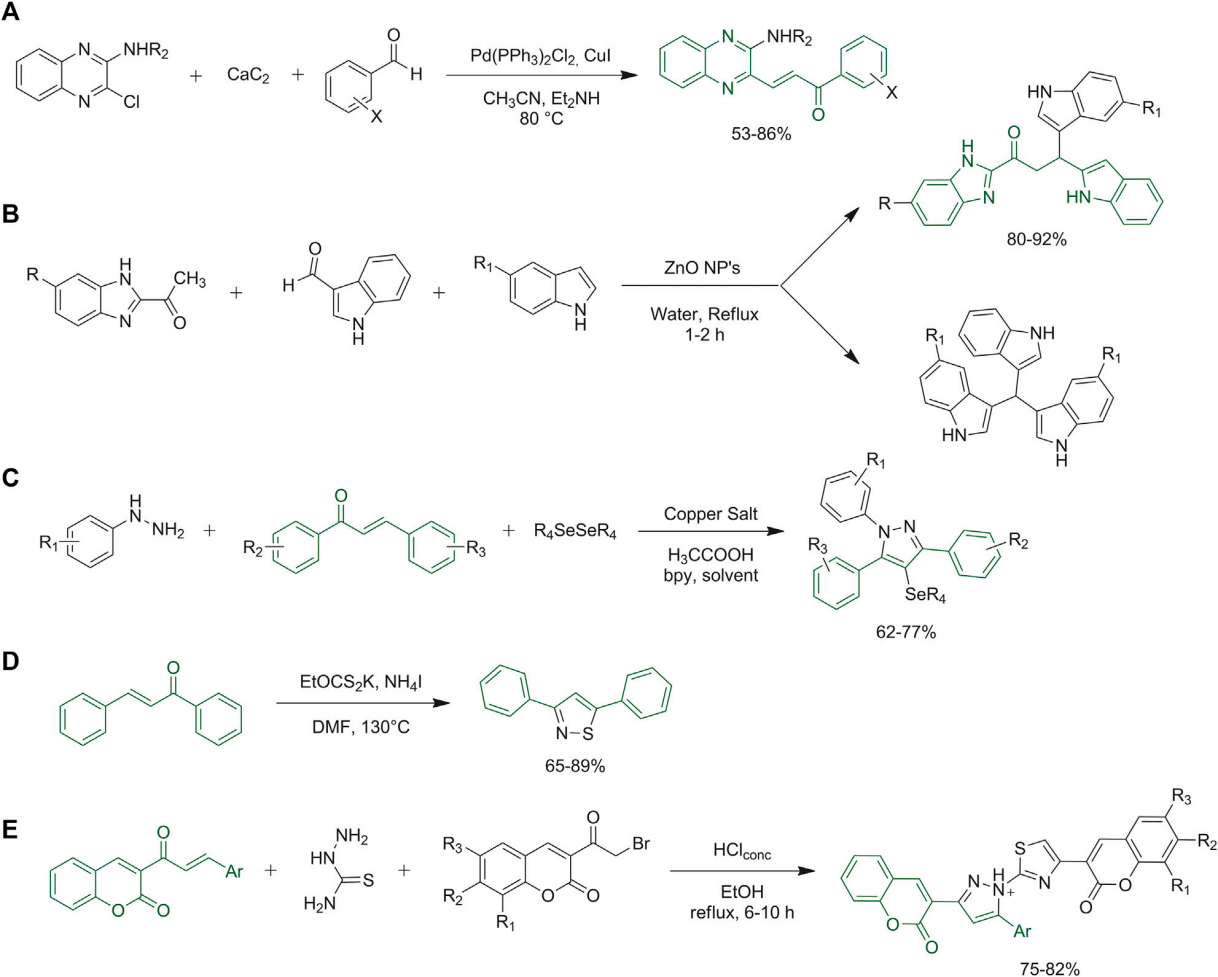
**(A)** Synthesis of **(E)**-3-(2-aminoquinoxalin-3-yl)-1-arylprop-2-en-1-one derivatives from 2-amino-substituted-3-chloroquinoxalines, calcium carbide, and aromatic aldehydes; **(B)** synthesis of indole-condensed benzimidazole chalcones via one-pot synthesis; **(C)** synthetic strategies for the synthesis of 1,3,5-triaryl-4-(arylselanyl)-pyrazoles through one-pot synthesis; **(D)** metal-free approach to get off various isothiazoles via three-component one-pot route: general synthesis; **(E)** synthetic strategy to obtain designed thiazolyl-pyrazole-biscoumarin via eco-friendly one-pot synthetic approach.

The chalcone chemical scaffold may be synthesized by classical one-pot Claisen-Schmidt condensation, where the desired aldehyde and ketone mixed under basic conditions render final chalcones. (Murugesan et al., 2017a; Murugesan et al., 2017b). They may also be obtained from alternative one-pot synthetic approaches. Soozani *et al.* reported the synthesis of quinoxaline chalcones via a one-step reaction of 3-substituted-2- chloroquinoxalines, and aromatic aldehydes with calcium carbide, in acetonitrile using diethylamine as the base and Pd/Cu as the catalyst (Figure 10A) (Soozani et al., 2017). The use of calcium carbide, to avoid protection and deprotection steps, results in an efficient and green one-pot procedure (Soozani et al., 2017). Dasari and collaborators developed a one-pot synthesis of variously functionalized indole-condensed benzimidazole chalcones. Several solvents, catalysts, and temperatures were evaluated to tune the best conditions to obtain the desired compounds with good yields, as reported in Figure 10B. The chalcone is formed as the first step of the proposed mechanism and then it reacts as an intermediate with the indole-derivative to produce the final compounds (Dasari et al., 2020).

Several examples of chalcone derivatization have also been developed. De Aquino *et al.* reported the synthesis of various 1,3,5-triaryl-4-(arylselanyl)-pyrazoles via a one-pot approach starting from properly designed chalcones and phenylhydrazines with the addition of acetic acid, diphenyl diselenide, CuBr, 2,2′-bipyridine (bpy) and solvent. 1,3,5-Triaryl-4-(arylselanyl)-pyrazoles were obtained with good yields via the optimized conditions, as reported in Figure 10C (de Aquino et al., 2018). Several syntheses of substituted isothiazoles are reported in the literature, starting from different building blocks. Li and collaborators proposed the synthesis of various isothiazoles through a one-step synthetic route starting from, among others, chalcone derivatives. They optimized the following procedure: NH_4_I, EtOCS_2_K and water were added to the desired chalcone in DMF, and the reaction was carried out at 130°C for 12 h (Figure 10D). Isothiazoles were obtained in high yields (Li et al., 2021). Mahmoodi and Ghodsi reported the synthesis of thiazolyl-pyrazole-biscoumarin derivatives by a one-pot three-component green cyclo-condensation of variously substituted coumarin chalcones, with thiosemicarbazide and 2-bromocoumarin in ethanol and a catalytic amount of HCl. Details are reported in Figure 10E (Mahmoodi and Ghodsi, 2017).

**FIGURE 11:**
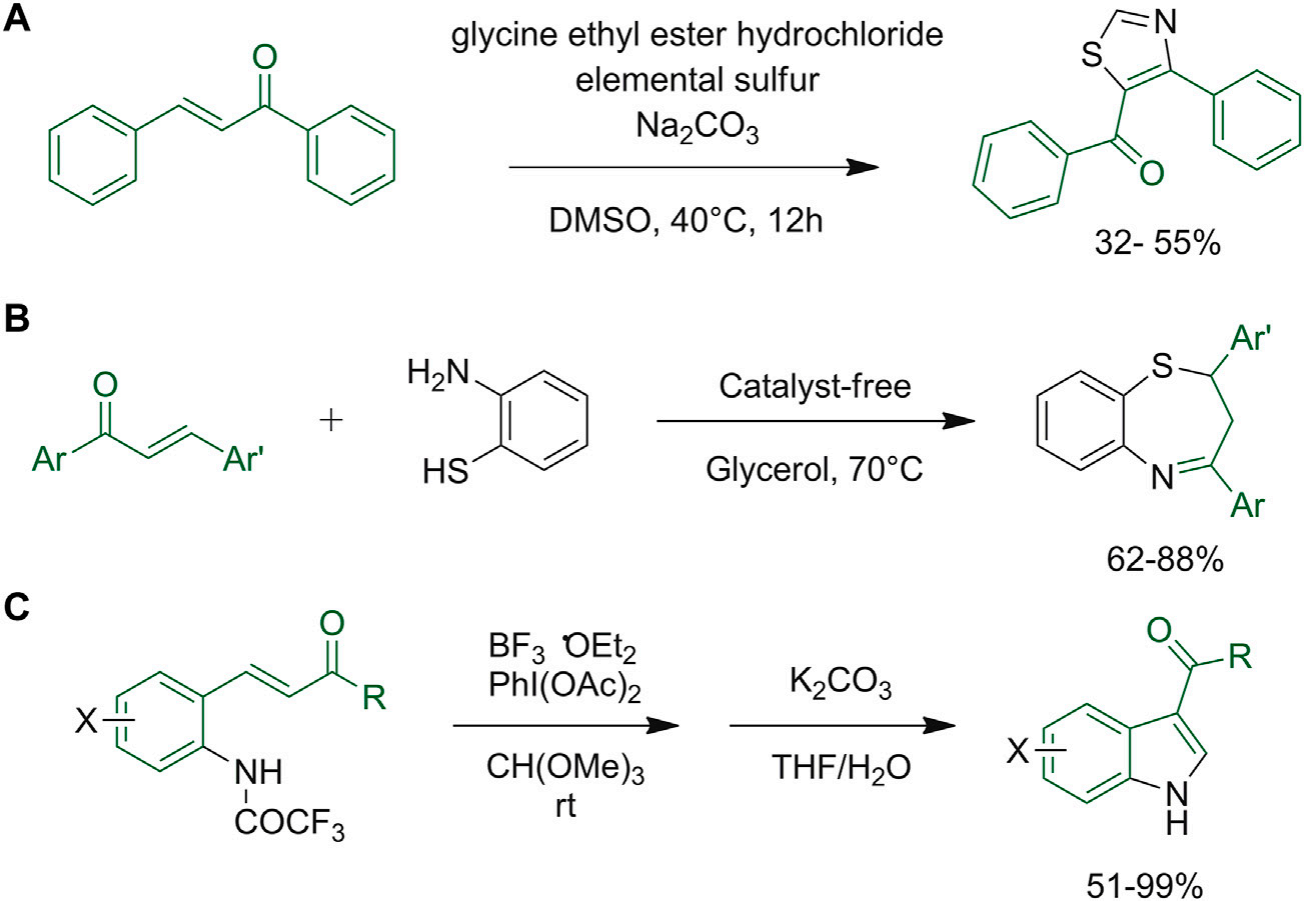
**(A)** Synthesis of 4,5-disubstituted thiazoles; **(B)** general synthetic protocol for the preparation of benzothiazepines via catalyst-free one-pot method using green glycerol medium; **(C)** one-pot synthesis of functionalized 3-acylindoles starting from properly selected chalcones.

Tran *et al.* reported a rare example of the synthesis of 4,5-disubsituted thiazoles in a one-pot reaction assembling chalcones, glycine ethyl ester hydrochloride and elemental sulfur (Tran et al., 2022). The research group also reported a plausible reaction mechanism, in which a trisulfur radical-anion, which is formed *in situ*, attacks the chalcone of interest, allowing the formation of a thiirane intermediate. Subsequently, the key enaminone intermediate is formed upon nucleophilic ring opening of the thiirane intermediate, at which point, the sulfuration of the activated alkene by excess elemental sulfur at high temperatures, rapid sequential aerobic oxidation and the final dealkoxycarbonylation induced by NaCl at high temperatures afford the final thiazole (Figure 11A). Yadav and collaborators synthesized benzothiazepines via a clean and efficient one-pot approach from chalcones and ortho-aminothiophenol (Yadav et al., 2019). Glycerol was selected as the medium to perform the synthesis under acid, base, or metal-free conditions. It resulted in a green synthesis with reusable media, short reaction time, catalyst-free conditions, easily available starting materials, broad substrate panel, easy process and work-up and final high yields (Yadav et al., 2019) (Figure 11B). 3-Acylindoles were obtained through oxidative rearrangement of 2-aminochalcone using a hypervalent iodine reagent optimizing one-pot conditions. The chalcone moiety is firstly functionalized as desired, and then cyclization of the indole scaffold is carried out under basic conditions in the same synthetic vessel. Optimized conditions (Figure 11C) afforded the desired products in quantitative yields (Nakamura et al., 2017).

Other examples of one-pot chalcone derivatizations are those reported by Ahmed *et al.*, which were aimed at synthesizing novel pyrimidine-based compounds. They developed a one-pot procedure where ketones and aldehydes were allowed to react to form the intermediate chalcone, then the properly functionalized guanidine was added to finalize the one-pot reaction, catalyzed in water media by green bio-organic Zn (l-proline)_2_ catalyst complex (Ahmed et al., 2021). Khalili and colleagues proposed graphene oxide (GO) as a catalyst for the sequential formation of chalcone and derivatization thereof by aza-Michael addition of the desired amines to form β-amino ketones (Khalili et al., 2020).

## 5 Green enabling technologies applied to the synthesis and derivatization of chalcones

### 5.1 Flow chemistry

Flow chemistry exploits a continuous flow reactor in which two pumps push the reagents, generating a continuous flowing stream where the reaction takes place. Different transformations can be achieved using different reactors such as coil, microchip, column, microspheres and tube-in-tube (TIT) (de Souza et al., 2018). The use of a flow reactor has several advantages, for instance, it is safer than the synthesis in round-bottomed flasks and reaction time is reduced, therefore, less energy is used. Moreover, gas substances can be used in flow reactions while being safely managed. Thanks to the capability of modifying and controlling parameters such as temperature, pressure and reactant concentration, it can be considered as a high-performance device to build up rapid, efficient and greener organic synthesis (de Souza et al., 2018). As detailed below, the flow chemistry technique has been recently applied to both the synthesis of heterocyclic chalcones and to the derivatization of chalcones into further functionalized analogues.

**FIGURE 12:**
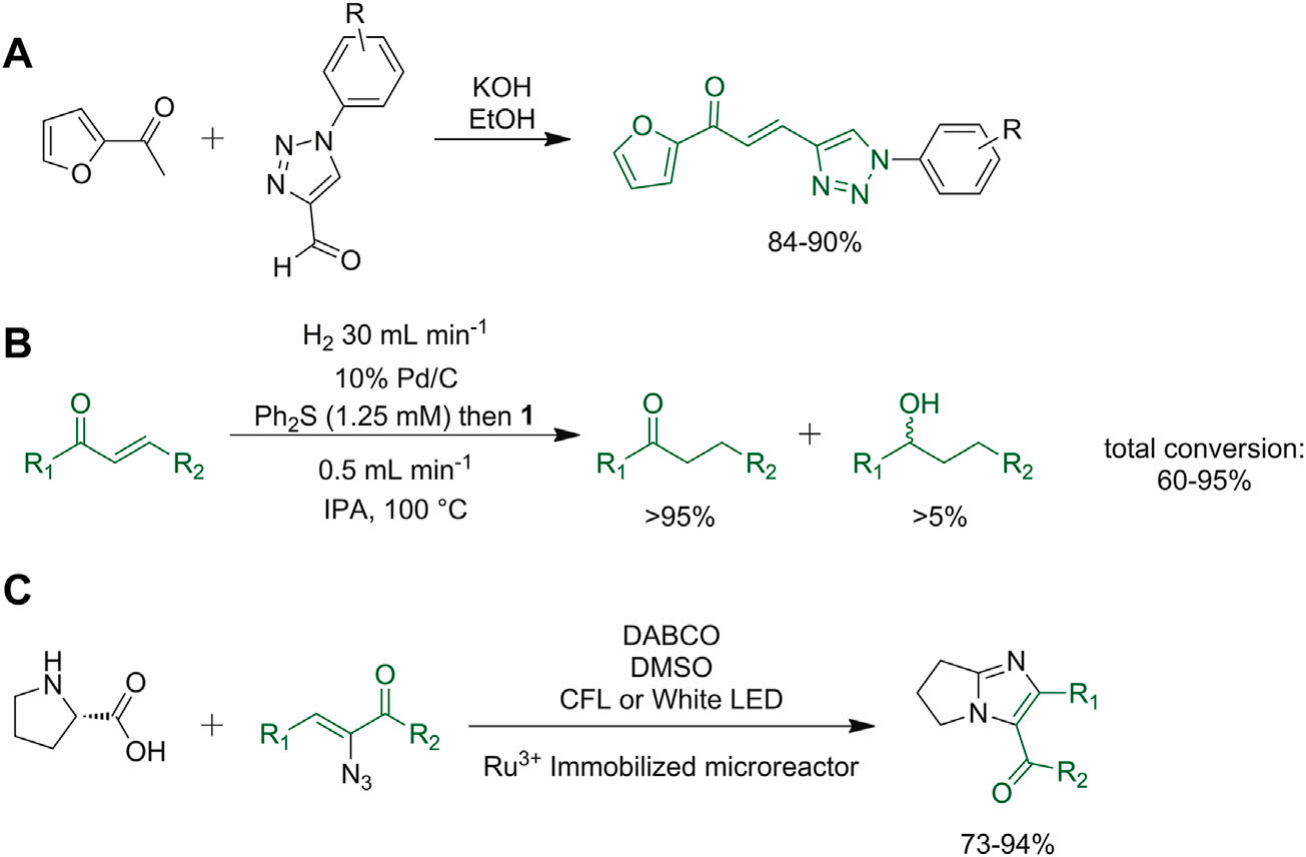
**(A)** Synthesis of 1,2,3-triazole-furan hybrid chalcone derivatives; **(B)** Pd/C-catalyzed reduction of chalcone moiety with diphenyl sulfide; **(C)** decarboxylation and a denitrogenation reaction to obtain imidazole derivatives.

Kumar *et al.* described the synthesis of 1,2,3-triazole-furan hybrid chalcone derivatives. The reaction depicted in Figure 12A was firstly carried out in batch, optimizing the best basic conditions. Then, the reaction was performed under microwave irradiation or in a flow reactor, thus attaining a shortening of the reaction time, and an improvement of the yield. The flow reactor device is favorable since it allows larger scale processes than microwave irradiation (Kumar et al., 2020). Moore and co-workers described a Pd/C-catalyzed reduction of chalcone moiety based on the addition of diphenyl sulfide that confers selectivity in conjugate reduction (Moore et al., 2019). This reduction reaction was adapted into a continuous hydrogenation method, based on the *in situ* constant production of hydrogen by water hydrolysis (Figure 12B). In order to avoid the poisoning of the catalyst caused by continuous pumping of sulfide additive that reduces the reactivity, pretreatment of the catalyst bed with a solution of diphenyl sulfide was performed (Moore et al., 2019). Adiyala *et al.* developed a novel synthetic strategy starting from *l* -proline and α-azidochalcones to obtain imidazole derivatives through decarboxylation and a denitrogenation reaction, respectively. A Ru^3+^-immobilized polydimethylsiloxane (PDMS) microreactor under fluorescent or white LED light was used for the continuous flow procedure. In this way, time was reduced under 2 min and yield increased up to 70–94% when compared to the reactions run in batch (Figure 12C) (Adiyala et al., 2020).

### 5.2 Photochemistry

In the last decades, photochemistry techniques had an exponential growth. The possibility of using visible light to activate photocatalysts or substrate-catalyst complexes makes this method an innovative and promising green approach. The growth in interest in its potential use is due to the need of mild reaction conditions. (Rehm, 2020). Photochemical chemistry uses photons as a reagent to generate radical intermediates during a reaction. Photons are the greenest reagents since no residues are left at the end of the reaction, even if the photon is either absorbed (and the reaction is activated) or not (and moves out of the vessel). Despite the described advantages, nowadays photochemistry finds poor applications both in academia and industries. Several reasons limit the integration of this technique in synthetic schemes, among which we can find the formation of undesired mixtures of products and the unpredictable course of the reactions together with the expensive devices (Wei and Cue, 2018).

Solar radiation is a green and renewable source of energy, useful for both photochemical and thermal reactions. CSR (concentrated solar radiation) can be obtained through the Fresnel lens and allows rapid organic synthesis, eventually reaching high temperatures and gaining final compounds in high yields. Jadhav and colleagues tested this new method by reacting 4-methoxyacetophenone with 4-fluorobenzaldehyde in the presence of potassium hydroxide (Jadhav et al., 2017). The advantage of using CSR instead of a classical reaction method is a short-term reaction with a higher yield. The solar radiations include infrared waves that allow the molecules to vibrate and rotate faster. Interestingly, Jadhav group proposed a possible explanation based on the bombardment radiations, giving an increment of energy, that speed up the reaction itself (Jadhav et al., 2017).

**FIGURE 13:**
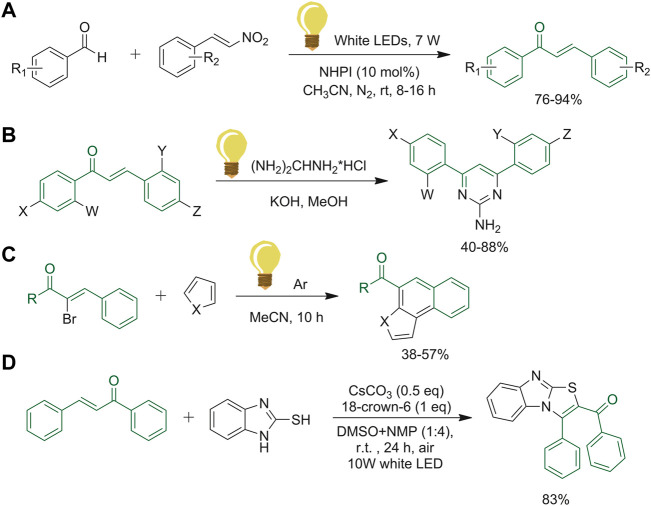
**(A)** Synthesis of chalcones with NHPI catalysts, an efficient visible light organophotocatalyst; **(B)** UV-radiation reaction procedure to afford pyrimidine derivatives; **(C)** photo-induced tandem vinyl radical cyclization with α-bromochalcone and pyrrole derivatives; **(D)** visible-light induced imidazo [2,1-*b*]thiazoles synthesis with photocatalysts-, oxidants- and transition metal-free technique.

The synthesis of substituted chalcones reported in Figure 13A was carried out by Tripathi *et al.* making the new and re-usable organo-photocatalyst, *N*-hydroxyphthalimide (NHPI), able to replace the metal-based visible light photocatalysts. These latter are efficient and productive, but could not be considered green catalysts (Tripathi et al., 2018). After the optimization of the reaction conditions, the compatibility of the synthetic methodology with different electron-donating and electron-withdrawing groups in the aromatic rings was assessed. Both gave high yields of the desired products, although the electron-withdrawing substituent allowed to achieve a faster reaction and afforded slightly higher yields (Tripathi et al., 2018). Saleem *et al.* conducted studies on pyrimidine scaffolds obtained from chalcone derivatives. They initiated with a conventional method, which includes the solubilization of the chalcone, guanidine HCl and the selected base in methanol. Subsequently, they performed the same reaction with UV-radiation instead of conventional heating (Saleem et al., 2018). The method illustrated in Figure 13B has been compared to the classic one, which showed an increment of yield and time reduction, giving the chance to perform the reaction in a more sustainable way (Saleem et al., 2018). In the last few years, many research groups have investigated the synthesis of polycyclic xanthones by photo-induced tandem vinyl radical cyclization. Yang and co-workers explored this reaction with α-bromo-chalcone and the respective pyrrole, as represented in Figure 13C (Yang et al., 2017). Under these conditions, the products of this one-pot reaction were obtained in a reasonable amount, and without the use of transition-metal complexes or high boiling solvent, making the procedure environment-friendly (Yang et al., 2017). Several imidazo [2,1-*b*]thiazoles were constructed by Chen and colleagues via electron-donor-acceptor (EDA) complex by performing a cyclization of chalcones and 2-mercaptobenzoimidazoles (Figure 13D). The aminothiolation reaction was performed through only visible-light irradiation. With this new technique, Chen *et al.* avoided the use of transition metals, external photocatalysts and oxidants (Chen et al., 2022).

## 6 Biocatalysis

Biocatalysis is considered a promising process in which enzymes are used to obtain pharmaceutical compounds. Among the main features of biocatalysis, selectivity, recyclability, an increase in atom economy, and safety are the most relevant. At the beginning, lipases and ketoreductases were the only available enzymes, now thanks to the development of technology for the production of new enzymes, it is possible to design an artificial “enzyme cascade” that allows to choose the desired proteins for the production of the target compounds (Carullo et al., 2019; Carullo et al., 2020; Mazzotta et al., 2021). Synthesis and modification of chalcones can be obtained using different biocatalytic approaches, whole-cell biocatalysts, free enzymes, or agro-food wastes (Bell et al., 2021).

An example of biocatalysis in the preparation of the chalcone scaffold is the one reported by Tamuli *et al.*, in which the use of fruit peal ash, as a green, easily available, and biodegradable catalyst for the Claisen-Schmidt condensation reaction allowed the formation of various chalcones and flavone derivatives. This reaction protocol can be carried out at room temperature with a reaction time that ranges from 10 min to 1 h, more importantly, is the possibility of carrying out the reaction with the use of green solvents or neat (Tamuli et al., 2020).

**FIGURE 14:**
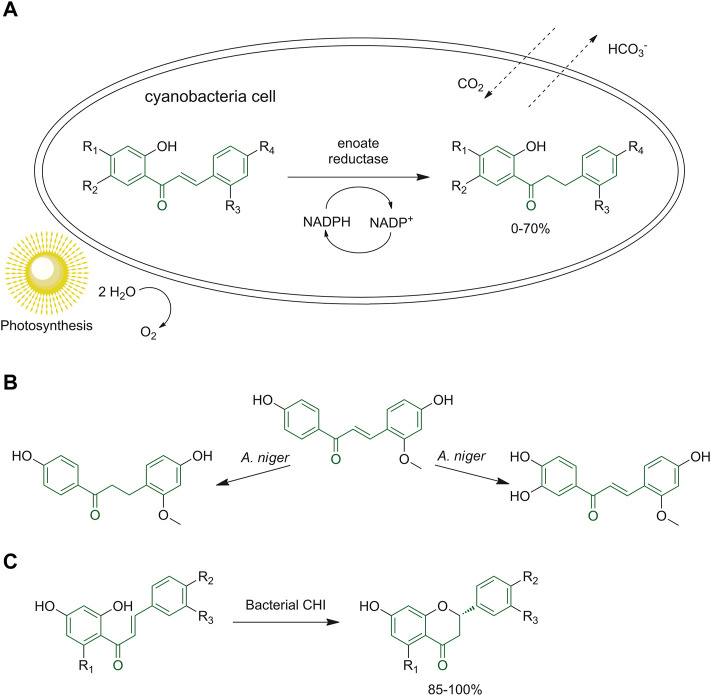
**(A)** General scheme of the biotransformation of methoxylated and methylated derivatives of 2′-hydroxychalcone in halophilic and freshwater cyanobacterial cells; **(B)** chalcone biotransformation by *A. niger;* **(C)** reaction performed by bacterial CHI to obtain (*S*)-flavones.

Żyszka-Haberecht *et al.* tested the introduction of methoxylated and methylated derivatives of 2′-hydroxychalcone in a culture of halophilic and freshwater cyanobacteria to verify the conditions of cells to the exposure of these molecules and the reactions occurring within the organism. They determined that the methoxylated chalcone derivatives were converted into the corresponding dihydro-derivatives as the only product (Figure 14A); instead, the methylated starting compounds gave dihydro- and hydroxy-, also ethoxy derivatives, highlighting how the methyl group can affect these biotransformations (Żyszka-Haberecht et al., 2019).

The ene-reductase (ER) enzyme is used to obtain pure enantiomers using asymmetric bioreduction of activated C=C double bonds when the stereogenic center is produced. Magallanes-Noguera and co-workers tried to expand the applications of ene-reductase enzymes, studying three axenic plant tissue cultures. *Tessaria absinthioides calli* demonstrated to have high selectivity for the C=C double bonds, *Medicago sativa* and *Capsicum annuum* were capable of hydrogenating also the carbonyl group (Magallanes-Noguera et al., 2017). Non-Conventional Yeasts (NCYs) were recently proven to express ER activities, thanks to the presence of cofactor-recycling systems for NAD(P)H useful for their cell metabolism. These ER activities can be exploited for whole-cells biotransformations in NCYs and have proven to be a cheap and useful alternative to purified enzymes to reduce the α,β-unsaturated chalcones. Dihydrochalcones were obtained from corresponding chalcones using a number of NCYs, such *Cyberlindnera amylophila*, *Kazachstania spencerorum*, *Naganishia diffluens*, *Kluyveromyces lactis*, among others, as reported by Filippucci *et al.* From the latter study, several lyophilized NCYs turned out as great alternatives with good reproducibility bioconversions, low deviations and high yields were also obtained (Filippucci et al., 2020).

Different biotransformation reactions can be achieved by *A. niger,* including hydroxylation, hydrogenation, epoxidation, hydrolysis, reduction, cyclization and alkylation, depending on the starting materials and the reaction conditions (Figure 14B). (Mohamed et al., 2022). Through the Sequence-Structure-Function-Evolution (SSFE) strategy the research group of Meinert and coworkers discovered 66 novel bacterial chalcone isomerase enzymes (CHIs) using the GenBank database. These enzymes have been investigated because they can be used as a sustainable synthetic route to obtain (*S*)-flavones starting from different hydroxylated and methoxylated (*S*)-chalcones (Figure 14C) (Meinert et al., 2021).

### 6.1 Artificial intelligence to design chalcone derivatives

Due to their broad spectrum of biological activities and therapeutic potential applications, the generation of focused libraries based on the chalcone scaffold related to different drug targets is a challenging task. To this end, in the new era of drug discovery, computational approaches are highly valuable, and not dispensable also for organic chemistry applications, thanks to the improvements in computing capacity, algorithms, and data availability. In fact, in silico methods based on AI could assist the synthesis of *ad hoc* compounds saving time, and money, and preventing damage to the environment (de Almeida et al., 2019; Jiménez-Luna et al., 2021). Considering the growing interest in the chalcone structural core, AI methods have been applied to prioritize derivatives to be synthesized, and here we report some successful approaches in this field of chalcones and derivatization products.

In the field of anti-infective agents, some studies have exploited AI to select and prioritize chalcone scaffolds. Gomes and coworkers employed these scaffolds to develop antitubercular agents. In particular, they developed a computational tool using matched molecular pair analysis (MMPA), starting from 604 chalcone derivatives with inhibition data against *Mycobacterium tuberculosis* strain H37Rv, for establishing and validating binary QSAR models. Fifteen different binary QSAR models were developed using several key descriptors such as MACCS, AtomPairs, Morgan, FeatMorgan, and Avalon fingerprints combined with machine learning methods (SVM, gradient boosting machine (GBM), and random forest (RF)). After that, they united all these models in a consensus ensemble model with significant statistical parameters (CCR = 0.77; Kappa = 0.53; Se = 0.79; Sp = 0.74; Coverage = 1.00), that was used in a screening of chalcone-based dataset, prioritizing 33 compounds for the synthesis. The synthesized compounds were evaluated for their antitubercular activity. Among them, 10 heteroaryl chalcone derivatives were found to exhibit nanomolar activities against replicating mycobacteria, low micromolar activity against nonreplicating bacteria, and nanomolar and micromolar activity against rifampicin- and isoniazid-resistant strains (Gomes et al., 2017b). The same research group, adopting a different computer-based approach, prioritized some compounds for the synthesis with potential anti-leishmanial activity. Starting from a chalcone-based library previously designed, they developed a target fishing protocol to identify possible activities against some leishmania drug targets. Using this computational protocol, nine most promising compounds and three potentially inactive compounds were experimentally evaluated against *Leishmania infantum* amastigotes and mammalian cells. Four synthesized compounds exhibited EC_50_ in the micromolar range, potentially acting as procathepsin L inhibitors. Furthermore, two chalcone-based derivatives, LabMol-65 and LabMol-73, showed low cytotoxicity and high selectivity toward Vero cells (Gomes et al., 2017a). Le and coworkers used a designed library containing chalcone-based molecules to identify, using ligand- and structure-based methods, possible molecules able to inhibit efflux pumps such as P-glycoprotein and NorA. In particular, by using machine learning techniques, they implemented a computational model using a total of 184 MOE 2D descriptors and 1,444 PaDEL 1D and 2D descriptors representing 63 different types of molecular properties, along with 166 MACCS (Molecular ACCess System) fingerprints, 881 PubChem fingerprints, and 307 substructure fingerprints to feature the compounds for the subsequent construction of classification, regression, and cognitive map models, respectively. Successivally, molecular docking studies employing the 3D structures of the efflux pumps provided the most performing compounds to synthesize. Based on this computer-based analysis, the authors identified four compounds (F29, F88, F90, and F91) potentially able to inhibit the selected efflux pumps. After the synthesis, the authors performed the validation of the identified hit compounds by *in vitro* tests, showing that F88 and F90 showed inhibitory potency against both transporters, with effectiveness against different *Staphylococcus aureus* strains overexpressing NorA and resistant to ciprofloxacin (Le et al., 2022).

Dong and collaborators described the synthesis of five chalcone-based derivatives endowed with a vasorelaxant profile using AI-based methods such as the support vector machine (SVM). This technique was used to generate a robust classification model employing 111 vasodilators and 232 non-vasodilators. The developed computational tool showed predictive accuracy of 93.0, 82.6, and 89.5% considering the training set, the test set and a validation set, respectively. Accordingly, they used the model for exploring the structure-activity relationship (SAR), identifying five promising chalcone-based derivatives classified as active as vasorelaxants. The selected target compounds were synthesized and evaluated for their vasorelaxant activity. Gratifyingly, all compounds showed a vasorelaxant profile in agreement with the prediction obtained employing the AI-based model (Dong et al., 2009).

Hwang and collaborators applied a computational protocol, mainly based on QSAR techniques and molecular docking, to perform a field-based rational design to synthesize a focused library of histone acetyltransferase inhibitor (HAT). In particular, starting from a previously developed HAT inhibitor, applying the *in silico* protocol, they found that the replacement of a moiety of the original inhibitor with a chalcone core improved the *in silico* performance of the conceived inhibitors. Accordingly, they synthesized some chalcone-based derivatives and among them, one compound showed the best inhibitory profile against p300 HAT in the submicromolar range. Because of the significant inhibitory potency, they performed a preliminary characterization of this chalcone derivative as an anti-fibrotic agent in both TGF-β1-stimulated lung fibroblasts and bleomycin-induced *in vivo* lung fibrosis mice. Delightfully, based on the obtained findings, the chalcone derivative should represent a good starting point for developing anti-fibrotic drugs (Hwang et al., 2020).

## 7 Conclusion

In the earlier phases of drug discovery, the hit and lead optimization cycles are extremely important and need to be accomplished in a timely fashion and in a highly efficient way in order to explore ample chemical space around selected scaffolds and/or privileged structures. For these and other reasons, metal-based C–C and C–X formation reactions represent a typical toolkit for medicinal chemists since the last 20 years. However, with the strong need of a paradigm shift toward the development of greener and more sustainable toolboxes, medicinal chemists are facing new challenges and bottlenecks in chemistry. Therefore, it is important to widen the range of green chemistry tools that could be useful for the preparation of biologically relevant structures. In this regard, seminal and recent literature reports prove that a growing interest is taking place in the development of sustainable chemistry applied to the chalcone scaffold. In particular, in the last few years, chalcones have been increasingly used as starting materials for further transformations to afford novel heterocyclic scaffolds endowed with potentially interesting biological activities and contributing to increasing the sustainable access to diverse scaffolds. Flow chemistry and biocatalysis are the most interesting techniques so far developed and show great potential for chalcones preparation and derivatization, but also photochemistry, despite some limitations such formation of complex mixtures or standardization of the reaction conditions could be a powerful technique that should be more in-depth investigated. Finally, the potential of AI for prioritizing compounds for the synthesis is another important aspect and from the examples reported in this review and considering the improvements that are expected in computational methods in the next future, we can anticipate that the *in silico* techniques will be necessary also for guiding the synthesis of focused libraries as in the presented cases of chalcone-based compounds. This will speed up the research on drug discovery, paying attention to the environment, reducing the experimental need.

## References

[B1] Adiyala P. R. Jang S. Vishwakarma N. K. Hwang Y.-H. Kim D.-P. (2020). Continuous-flow photo-induced decarboxylative annulative access to fused imidazole derivatives via a microreactor containing immobilized ruthenium. Green Chem. 22, 1565–1571. 10.1039/c9gc03496j

[B2] Adole V. A. Jagdale B. S. Pawar T. B. Sagane A. A. (2020). Ultrasound promoted stereoselective synthesis of 2, 3-dihydrobenzofuran appended chalcones at ambient temperature. S. Afr. J. Chem. 73, 35–43. 10.17159/0379-4350/2020/v73a6

[B3] Aegurla B. Peddinti R. K. (2018). Metal-free sulfonylation of α, β-conjugated systems by using sulfonyl hydrazides. Asian J. Org. Chem. 7, 946–954. 10.1002/ajoc.201700696

[B4] Ahmed E. A. Soliman A. M. M. Ali A. M. Ali El-Remaily M. A. E. A. A. (2021). Boosting the catalytic performance of zinc linked amino acid complex as an eco-friendly for synthesis of novel pyrimidines in aqueous medium. Appl. Organomet. Chem. 35, e6197. 10.1002/aoc.6197

[B5] Antony J. Rappai J. P. Ramakrishnan K. Natarajan R. (2021). Aldonitrones as aldehyde surrogates in solvent free synthesis of chalcones under mechanochemical activation. Results Chem. 3, 100224. 10.1016/j.rechem.2021.100224

[B6] Arafa W. A. A. (2018). Sustainable catalytic process with a high eco-scale score for the synthesis of novel series of bischalcones through claisen-schmidt condensation. J. Heterocycl. Chem. 55, 456–464. 10.1002/jhet.3063

[B7] Bahekar S. P. Agrawal N. R. Sarode P. B. Agrawal A. R. Chandak H. S. (2017). L-proline nitrate: An amino acid ionic liquid for green and efficient conjugate addition of thiols to sulfonamide chalcones. ChemistrySelect 2, 9326–9329. 10.1002/slct.201701891

[B8] Bahrami K. Khodaei M. M. Batooie N. Hosseinzadeh N. Foroumadi A. (2019). Hexyltriphenylphosphonium bromide as an absolutely chemoselective ionic liquid catalyst in the three-component reaction of aryl aldehydes, acetophenones and malononitrile. ChemistrySelect 4, 6190–6193. 10.1002/slct.201901076

[B9] Balyan S. Sharma R. Lal J. (2020). Microwave-assisted, efficient and eco-friendly synthesis of novel 3H-Benzo[b] [1, 4]diazepine derivatives using basic alumina as a reusable catalyst. Chem. Afr. 3, 35–44. 10.1007/s42250-019-00110-w

[B10] Bell E. L. Finnigan W. France S. P. Hepworth L. J. Lovelock S. L. Hayes M. A. (2021). Biocatalysis. Nat. Rev. Methods Prim. 1, 46–21. 10.1038/s43586-021-00044-z

[B11] Carullo G. Governa P. Leo A. Gallelli L. Citraro R. Cione E. (2019). Quercetin-3-Oleate contributes to skin wound healing targeting FFA1/GPR40. ChemistrySelect 4 (29), 8429–8433. 10.1002/slct.201902572

[B12] Carullo G. Mazzotta S. Koch A. Hartmann K. M. Friedrich O. Gilbert D. F. (2020). New oleoyl hybrids of natural antioxidants: Synthesis and *in vitro* evaluation as inducers of apoptosis in colorectal cancer cells. Antioxidants 9, 1077. 10.3390/antiox9111077 PMC7692320 33153029

[B13] Chan C.-K. Lai C.-Y. Wang C.-C. (2020). Environmentally friendly nafion-mediated friedlander quinoline synthesis under microwave irradiation: Application to one-pot synthesis of substituted quinolinyl chalcones. Synth. (Stuttg) 52, 1779–1794. 10.1055/s-0039-1690088

[B14] Chaudhari P. P. Rajput S. S. (2018). Clean synthesis and antimicrobial interpretation of azo (dipyrano) and bis- chalcones derivatives from n-phenyl pyrrolidine-2, 5-dione and n-phenyl piperidine-2, 6-dione. Heterocycl. Lett. 8, 133–144.

[B15] Chen Z. Xue F. Zhang Y. Jin W. Wang B. Xia Y. (2022). Visible-light-promoted [3 + 2] cyclization of chalcones with 2-mercaptobenzimidazoles: A protocol for the synthesis of imidazo[2, 1- b]thiazoles. Org. Lett. 24, 3149–3154. 10.1021/acs.orglett.2c00867 35451846

[B16] Cho S. Kim S. Jin Z. Yang H. Han D. Baek N. I. (2011). Isoliquiritigenin, a chalcone compound, is a positive allosteric modulator of GABA A receptors and shows hypnotic effects. Biochem. Biophys. Res. Commun. 413, 637–642. 10.1016/j.bbrc.2011.09.026 21945440

[B17] Cuellar J. E. Quiñones W. Robledo S. Gil J. Durango D. (2022). Coumaro-chalcones synthesized under solvent-free conditions as potential agents against malaria, leishmania and trypanosomiasis. Heliyon 8, e08939. 10.1016/j.heliyon.2022.e08939 35198789 PMC8851253

[B18] Das S. Porashar B. Saikia S. Borah R. (2020). Bronsted acidic ionic liquids catalysed sequential michael-like addition of indole with chalcones via claisen-schmidt condensation. ChemistrySelect 5, 3041–3047. 10.1002/slct.201904851

[B19] Dasari G. K. Sunkara S. Gadupudi P. C. R. (2020). Green and ecofriendly synthesis of indole-condensed benzimidazole chalcones in water and their antimicrobial evaluations. J. Heterocycl. Chem. 57, 1201–1210. 10.1002/jhet.3856

[B20] de Almeida A. F. Moreira R. Rodrigues T. (2019). Synthetic organic chemistry driven by artificial intelligence. Nat. Rev. Chem. 3, 589–604. 10.1038/s41570-019-0124-0

[B21] de Aquino T. F. B. Seidel J. P. de Oliveira D. H. do Nascimento J. E. R. Alves D. Perin G. (2018). Copper-catalyzed synthesis of 1, 3, 5-triaryl-4-(organylselanyl)-1H-pyrazoles by one-pot multicomponent reactions. Tetrahedron Lett. 59, 4090–4095. 10.1016/j.tetlet.2018.10.008

[B22] De La Hoz A. Díaz-Ortiz A. Prieto P. (2016). “Microwave-assisted green organic synthesis,” in Alternative energy sources for green chemistry (The Royal Society of Chemistry). 10.1039/9781782623632-00001

[B23] de Souza J. M. Galaverna R. de Souza A. A. N. Brocksom T. J. Pastre J. C. de Souza R. O. M. A. (2018). Impact of continuous flow chemistry in the synthesis of natural products and active pharmaceutical ingredients. An. Acad. Bras. Cienc. 90, 1131–1174. 10.1590/0001-3765201820170778 29873673

[B24] Devkate C. G. Kola S. S. Gaikwad D. D. Siddique M. I. M. (2018). Ultrasound promoted one pot synthesis of 1, 5-benzothiazepines using polyethylene glycol (PEG-400). Int. Res. J. Pharm. 9, 182–185. 10.7897/2230-8407.0911280

[B25] Dhadda S. Goswami P. G. Sharma H. (2022). “Green synthesis of chalcone derivatives using chalcones as precursor,” in Green chemistry - new perspectives (London: IntechOpen). 10.5772/intechopen.103959

[B26] Dong X. Chen J. Jiang C. Liu T. Hu Y. (2009). Design, synthesis, and biological evaluation of prenylated chalcones as vasorelaxant agents. Arch. Pharm. Weinh. 342, 428–432. 10.1002/ardp.200800229 19544479

[B27] Draye M. Chatel G. Duwald R. (2020). Ultrasound for drug synthesis: A green approach. Pharmaceuticals 13, 23. 10.3390/ph13020023 PMC7168956 32024033

[B28] Duran N. Polat M. F. Aktas D. A. Alagoz M. A. Ay E. Cimen F. (2021). New chalcone derivatives as effective against SARS-CoV-2 agent. Int. J. Clin. Pract. 75 (12), e14846. 10.1111/ijcp.14846 34519118 PMC8646589

[B29] Filippucci S. Tasselli G. Kenza Labbani F.-Z. Turchetti B. Cramarossa M. R. Buzzini P. (2020). Non-conventional Yeasts as sources of ene-reductases for the bioreduction of chalcones. Fermentation 6, 29. 10.3390/fermentation6010029

[B30] Gomes C. Vinagreiro C. S. Damas L. Aquino G. Quaresma J. Chaves C. (2020). Advanced mechanochemistry device for sustainable synthetic processes. ACS Omega 5, 10868–10877. 10.1021/acsomega.0c00521 32455207 PMC7240818

[B31] Gomes M. N. Alcântara L. M. Neves B. J. Melo-Filho C. C. Freitas-Junior L. H. Moraes C. B. (2017a). Computer-aided discovery of two novel chalcone-like compounds active and selective against Leishmania infantum. Bioorg. Med. Chem. Lett. 27, 2459–2464. 10.1016/j.bmcl.2017.04.010 28434763 PMC6020026

[B32] Gomes M. N. Braga R. C. Grzelak E. M. Neves B. J. Muratov E. Ma R. (2017b). QSAR-driven design, synthesis and discovery of potent chalcone derivatives with antitubercular activity. Eur. J. Med. Chem. 137, 126–138. 10.1016/j.ejmech.2017.05.026 28582669 PMC6031314

[B33] Gomes M. N. Muratov E. N. Pereira M. Peixoto J. C. Rosseto L. P. Cravo P. V. L. (2017c). Chalcone derivatives: Promising starting points for drug design. Molecules 22, 1210. 10.3390/molecules22081210 PMC6152227 28757583

[B34] Gupta S. Ameta C. Ameta R. Punjabi P. B. (2020). “Click chemistry: A tool for green chemical organic synthesis,” in Green sustainable process for chemical and environmental engineering and science (Elsevier), 13–48. 10.1016/B978-0-12-819539-0.00002-6

[B35] Hajipour A. R. Khorsandi Z. (2017). Application of immobilized proline on CNTs and proline ionic liquid as novel organocatalysts in the synthesis of 2-amino-4H-pyran derivatives: A comparative study between their catalytic activities. ChemistrySelect 2, 8976–8982. 10.1002/slct.201700847

[B36] Higuchi K. Watanabe T. Tanigawa T. Tominaga K. Fujiwara Y. Arakawa T. (2010). Sofalcone, a gastroprotective drug, promotes gastric ulcer healing following eradication therapy for *Helicobacter pylori*: A randomized controlled comparative trial with cimetidine, an H2-receptor antagonist. J. Gastroenterol. Hepatol. 25, 155–160. 10.1111/j.1440-1746.2010.06232.x 20586860

[B37] Hwang S. Y. Park S. Y. Hong J. Y. Lee S. Y. Shin J. H. Na Y. (2020). Field-based rational design of p300 histone acetyltransferase inhibitor and systematic evaluation as an anti-fibrotic agent. Chem. Commun. 56, 9795–9798. 10.1039/d0cc03553j 32701101

[B38] Iwamura C. Shinoda K. Yoshimura M. Watanabe Y. Obata A. Nakayama T. (2010). Naringenin chalcone suppresses allergic asthma by inhibiting the type-2 function of CD4 T cells. Allergol. Int. 59, 67–73. 10.2332/allergolint.09-OA-0118 20035147

[B39] Jadhav N. L. Pandit A. B. Pinjari D. V. (2017). Green approach for the synthesis of chalcone (3-(4-fluorophenyl)-1-(4-methoxyphenyl)prop-2-en-1-one) using concentrated solar radiation. Sol. Energy 147, 232–239. 10.1016/j.solener.2017.03.047

[B40] Janković T. Turković N. Kotur-Stevuljević J. Vujić Z. Ivković B. (2020). Differences in antioxidant potential of chalcones in human serum: *In vitro* study. Chem. Biol. Interact. 324, 109084. 10.1016/j.cbi.2020.109084 32289290

[B41] Jasril J. Teruna H. Y. Aisyah A. Nurlaili N. Hendra R. (2019). Microwave assisted synthesis and evaluation of toxicity and antioxidant activity of pyrazoline derivatives. Indones. J. Chem. 19, 583–591. 10.22146/ijc.34285

[B42] Jiménez-Luna J. Grisoni F. Weskamp N. Schneider G. (2021). Artificial intelligence in drug discovery: Recent advances and future perspectives. Expert Opin. Drug Discov. 16, 949–959. 10.1080/17460441.2021.1909567 33779453

[B43] Kakade G. K. Vedpathak S. G. (2020). Ultrasound assisted green synthesis of 2-furan-2-yl-4H-chromen-4-ones from chalcones. Int. J. Curr. Pharm. Res. 12, 84–86. 10.22159/ijcpr.2020v12i3.38312

[B44] Kalluraya B. Mallya S. Kumar K A. (2018). Microwave assisted neat synthesis of spiropyrrolidine library. J. Heterocycl. Chem. 55, 2075–2081. 10.1002/jhet.3247

[B45] Kannan D. Naveen S. Jagadeesan G. Lokanath N. K. Thennarasu S. (2019). Ultrasonic cavitation facilitates rapid synthesis of trisubstituted pyrazole scaffolds through Michael addition/domino cyclization. ChemistrySelect 4, 9807–9810. 10.1002/slct.201902126

[B46] Karimi-Jaberi Z. Masoudi B. Rahmani A. Alborzi K. (2020). Triethylammonium hydrogen sulfate [Et3NH] [HSO4] as an efficient ionic liquid catalyst for the synthesis of coumarin derivatives. Polycycl. Aromat. Compd. 40, 99–107. 10.1080/10406638.2017.1363061

[B47] Kaur H. Singh R. Kant R. (2022). Synthesis, molecular docking, and antitubercular evaluation of triazole–chalcone conjugates. Russ. J. Org. Chem. 58, 518–525. 10.1134/S107042802204008X

[B48] Khalili D. Lavian S. Moayyed M. (2020). Graphene oxide as a catalyst for one-pot sequential aldol coupling/aza-Michael addition of amines to chalcones through *in situ* generation of Michael acceptors under neat conditions. Tetrahedron Lett. 61, 151470. 10.1016/j.tetlet.2019.151470

[B49] Kothandapani J. Megarajan S. Ayaz Ahmed K. B. Priyanka M. Anbazhagan V. Selva Ganesan S. (2017). Stearyl MethoxyPEGglycol succinate-A designer micellar medium for diverse aniline derivatives synthesis. ACS Sustain. Chem. Eng. 5, 5740–5745. 10.1021/acssuschemeng.7b00317

[B50] Kumar K. S. Siddaiah V. Lilakar J. D. Ganesh A. (2020). An efficient continuous-flow synthesis and evaluation of antimicrobial activity of novel 1, 2, 3-Triazole-Furan hybrid chalcone derivatives. Chem. Data Collect. 28, 100457. 10.1016/j.cdc.2020.100457

[B51] La Sorella G. Strukul G. Scarso A. (2015). Recent advances in catalysis in micellar media. Green Chem. 17, 644–683. 10.1039/C4GC01368A

[B52] Le M. T. Trinh D. T. T. Ngo T. Du Tran-Nguyen V. K. Nguyen D. N. Hoang T. (2022). Chalcone derivatives as potential inhibitors of P-glycoprotein and NorA: An *in silico* and *in vitro* study. Biomed. Res. Int. 2022, 1–9. 10.1155/2022/9982453 PMC8976639 35378788

[B53] Li J. Li J. Ji X. Liu Q. Chen L. Huang Y. (2021). Transition metal‐free synthesis of substituted isothiazoles via three-component annulation of alkynones, xanthate and NH 4 I. Adv. Synth. Catal. 363, 1059–1068. 10.1002/adsc.202001179

[B54] Li Z. Zhao H. Han H. Liu Y. Song J. Guo W. (2017). Graphene-supported ZnO nanoparticles: An efficient heterogeneous catalyst for the Claisen-Schmidt condensation reaction without additional base. Tetrahedron Lett. 58, 3984–3988. 10.1016/j.tetlet.2017.09.011

[B55] Magallanes-Noguera C. Cecati F. M. Mascotti M. L. Reta G. F. Agostini E. Orden A. A. (2017). Plant tissue cultures as sources of new ene- and ketoreductase activities. J. Biotechnol. 251, 14–20. 10.1016/j.jbiotec.2017.03.023 28359867

[B56] Mahapatra D. Bharti S. Asati V. (2017). Chalcone derivatives: Anti-inflammatory potential and molecular targets perspectives. Curr. Top. Med. Chem. 17, 3146–3169. 10.2174/1568026617666170914160446 28914193

[B57] Mahapatra D. K. Bharti S. K. Asati V. (2015). Chalcone scaffolds as anti-infective agents: Structural and molecular target perspectives. Eur. J. Med. Chem. 101, 496–524. 10.1016/j.ejmech.2015.06.052 26188621

[B58] Mahato S. Santra S. Chatterjee R. Zyryanov G. V. Hajra A. Majee A. (2017). Bronsted acidic ionic liquid-catalyzed tandem reaction: An efficient approach towards regioselective synthesis of pyrano[3, 2-c]coumarins under solvent-free conditions bearing lower E-factors. Green Chem. 19, 3282–3295. 10.1039/c7gc01158j

[B59] Mahmoodi N. O. Ghodsi S. (2017). Thiazolyl-pyrazole-biscoumarin synthesis and evaluation of their antibacterial and antioxidant activities. Res. Chem. Intermed. 43, 661–678. 10.1007/s11164-016-2644-2

[B60] Mahurkar S. S. Makone S. S. More R. A. (2019). An efficient and recyclable catalyst for synthesis of 1, 3-diphenyl-3-(phenyl thio). Chem. Biol. Interface 9, 277–284.

[B61] Mazzotta S. Governa P. Borgonetti V. Marcolongo P. Nanni C. Gamberucci A. (2021). Pinocembrin and its linolenoyl ester derivative induce wound healing activity in HaCaT cell line potentially involving a GPR120/FFA4 mediated pathway. Bioorg. Chem. 108, 104657. 10.1016/j.bioorg.2021.104657 33556697

[B62] Meinert H. Yi D. Zirpel B. Schuiten E. Geißler T. Gross E. (2021). Discovery of novel bacterial chalcone isomerases by a sequence-structure-function-evolution strategy for enzymatic synthesis of (S)-Flavanones. Angew. Chem. Int. Ed. 60, 16874–16879. 10.1002/anie.202107182 PMC8361940 34129275

[B63] Mishra A. Rai P. Pandey Y. K. Singh J. Singh J. (2017). An eco-sustainable synthetic approach for 4, 5-dihydro-1H-pyrazoles via DBU catalysis in micellar medium. ChemistrySelect 2, 10979–10983. 10.1002/slct.201702400

[B64] Mohamed T. A. Ali S. K. Elshamy A. I. Saleh I. A. Ibrahim M. A. A. Atia M. A. M. (2022). Plant cell cultures: An enzymatic tool for polyphenolic and flavonoid transformations. Phytomedicine 100, 154019. 10.1016/j.phymed.2022.154019 35325826

[B65] Moore J. C. Howie R. A. Bourne S. L. Jenkins G. N. Licence P. Poliakoff M. (2019). *In situ* sulfidation of Pd/C: A straightforward method for chemoselective conjugate reduction by continuous hydrogenation. ACS Sustain. Chem. Eng. 7, 16814–16819. 10.1021/acssuschemeng.9b04347

[B66] Mubarak S. Zia-Ur-Rehman M. Jamil N. Zaheer M. Arshad M. N. Asiri A. M. (2019). Environment friendly synthesis of N’-(1, 3-diphenylallylidene)-1-ethyl-7-methyl-4-oxo-1, 4-dihydro-1, 8-naphthyridine-3-carbohydrazides: Crystal structure and their anti-oxidant potential. Chem. Pharm. Bull. 67, 1191–1200. 10.1248/cpb.c19-00478 31685748

[B67] Murugesan A. Gengan R. M. Lin C.-H. (2017a). Efficient synthesis of ethyl-piperazinyl quinolinyl-(E)-chalcone derivatives via Claisen-Schmidt reaction by using TiO2-BPTETSA catalyst. J. Taiwan Inst. Chem. Eng. 80, 852–866. 10.1016/j.jtice.2017.07.014

[B68] Murugesan A. Gengan R. M. Rajamanikandan R. Ilanchelian M. Lin C.-H. (2017b). One-pot synthesis of Claisen-Schmidt reaction through (E)-chalcone derivatives: Spectral studies in human serum albumin protein binding and molecular docking investigation. Synth. Commun. 47, 1884–1904. 10.1080/00397911.2017.1355466

[B69] Muthuvel I. Thirunarayanan G. Thangaraj V. Sundaramurthy N. Rajalakshmi S. Usha V. (2021). Study of catalytic activity of Zn3(PO4)2 on the synthesis of some pyrenyl enones. Mater. Today Proc. 43, 2203–2207. 10.1016/j.matpr.2020.12.169

[B70] Nakamura A. Tanaka S. Imamiya A. Takane R. Ohta C. Fujimura K. (2017). Synthesis of 3-acylindoles by oxidative rearrangement of 2-aminochalcones using a hypervalent iodine reagent and cyclization sequence. Org. Biomol. Chem. 15, 6702–6705. 10.1039/c7ob01536d 28749517

[B71] Ouyang Y. Li J. Chen X. Fu X. Sun S. Wu Q. (2021). Chalcone derivatives: Role in anticancer therapy. Biomolecules 11, 894. 10.3390/biom11060894 34208562 PMC8234180

[B72] Patel T. Gaikwad R. Jain K. Ganesh R. Bobde Y. Ghosh B. (2019). First report on 3-(3-oxoaryl) indole derivatives as anticancer agents: Microwave assisted synthesis, *in vitro* screening and molecular docking studies. ChemistrySelect 4, 4478–4482. 10.1002/slct.201900088

[B73] Pereira D. Goncalves C. Martins B. T. Palmeira A. Vasconcelos V. Pinto M. (2021). Flavonoid glycosides with a triazole moiety for marine antifouling applications: Synthesis and biological activity evaluation. Mar. Drugs 19, 5. 10.3390/md19010005 PMC7823860 33374188

[B74] Pogaku V. Krishna V. S. Sriram D. Rangan K. Basavoju S. (2019). Ultrasonication-ionic liquid synergy for the synthesis of new potent anti-tuberculosis 1, 2, 4-triazol-1-yl-pyrazole based spirooxindolopyrrolizidines. Bioorg. Med. Chem. Lett. 29, 1682–1687. 10.1016/j.bmcl.2019.04.026 31047752

[B75] Pozzetti L. Ibba R. Rossi S. Taglialatela-Scafati O. Taramelli D. Basilico N. (2022). Total synthesis of the natural chalcone lophirone E, synthetic studies toward benzofuran and indole-based analogues, and investigation of anti-leishmanial activity. Molecules 27, 463. 10.3390/molecules27020463 35056779 PMC8778746

[B76] Prabhakar M. Merry T. Zatsu R. Tsurho S. Merugu R. (2017). Microwave-assisted fast and efficient green synthesis of 9-anthracenyl chalcones and their anti-bacterial activity. IOSR J. Pharm. 7, 24–32.

[B77] Praveena P. Sarojini B. K. Madan Kumar S. (2019). Mechanochemical synthesis and characterizations of chalcone derivatives: (2E)-3-[4-(Benzyloxy)phenyl]-1-(thiophen-2-yl)prop ‑2-en-1-one and (2e)-3-(anthracen-9-yl)-1- (thiophen-2-yl)prop-2-en-1-one. Chem. Data Collect. 24, 100298. 10.1016/j.cdc.2019.100298

[B78] Rahim A. Bhuiyan M. M. H. Matin M. M. Ali R. Kabir E. (2018). Synthesis of 2-Phenylchromen-4-one derivatives by conventional and microwave: Assisted techniques, and their antimicrobial evaluation. Int. J. Chem. Stud. 6, 1–4.

[B79] Rajaguru K. Mariappan A. Muthusubramanian S. Bhuvanesh N. (2017). Divergent reactivity of α-azidochalcones with metal β-diketonates: Tunable synthesis of substituted pyrroles and indoles. Org. Chem. Front. 4, 124–129. 10.1039/c6qo00541a

[B80] Ranganathan K. Kamalakkannan D. Suresh R. Sakthinathan S. P. Arulkumaran R. Sundararajan R. (2020). Cu2+/Zeolite catalyzed aldol condensation: Greener synthesis of 4’-piperidinophenyl enones. Mater. Today Proc. 22, 1196–1199. 10.1016/j.matpr.2019.12.120

[B81] Rehm T. H. (2020). Flow photochemistry as a tool in organic synthesis. Chem. Eur. J. 26, 16952–16974. 10.1002/chem.202000381 32427387 PMC7821313

[B82] Rizk S. A. El-Hashash M. A. El-Badawy A. A. (2017). Ultrasonic and grinding aptitudes of one-pot synthesis of 5-(4-chlorophenyl)-7-(3, 4-dimethyl phenyl)-2-oxo-2H-Pyrano[2, 3-b]Pyridine derivatives as antibacterial agents. J. Heterocycl. Chem. 54, 2003–2011. 10.1002/jhet.2797

[B83] Rocha D. H. A. Vaz P. A. A. M. Pinto D. C. G. A. Silva A. M. S. (2019). Synthesis chalones and their isomerization into flavanones and azaflavanones. Methods Protoc. 2, 70. 10.3390/mps2030070 PMC6789463 31443245

[B84] Rosa G. P. Seca A. M. L. Barreto M. D. C. Pinto D. C. G. A. (2017). Chalcone: A valuable scaffold upgrading by green methods. ACS Sustain. Chem. Eng. 5, 7467–7480. 10.1021/acssuschemeng.7b01687

[B85] Sahoo B. M. Banik B. K. MazaharunnisaRao N. S. Raju B. (2019). Microwave assisted green synthesis of benzimidazole derivatives and evaluation of their anticonvulsant activity. Curr. Microw. Chem. 6, 23–29. 10.2174/2213335606666190429124745

[B86] Sahoo B. M. Sahoo B. Panda J. Kumar A. (2017). Microwave-induced synthesis of substituted isoxazoles as potential antimicrobial agents. Curr. Microw. Chem. 4, 146–151. 10.2174/2213335603666160926101734

[B87] Sakirolla R. Tadiparthi K. Yaeghoobi M. Abd Rahman N. (2018). Di-cationic ionic liquid catalyzed synthesis of 1, 5-benzothiazepines. Asian J. Chem. 30, 107–115. 10.14233/ajchem.2018.20920

[B88] Saleem S. Nazli Z.-H. Saleem N. Bashir M. S. Hussain S. Parveen B. (2018). Synthesis, spectroscopy and biological studies of chalcone derived pyrimidines. Pharma Chem. 10, 110–117.

[B89] Salehi B. Quispe C. Chamkhi I. El Omari N. Balahbib A. Sharifi-Rad J. (2021). Pharmacological properties of chalcones: A review of preclinical including molecular mechanisms and clinical evidence. Front. Pharmacol. 11, 592654. 10.3389/fphar.2020.592654 33536909 PMC7849684

[B90] Shankaraiah N. Sakla A. P. Laxmikeshav K. Tokala R. (2020). Reliability of click chemistry on drug discovery: A personal account. Chem. Rec. 20, 253–272. 10.1002/tcr.201900027 31419056

[B91] Sharma M. K. Parashar S. Chahal M. Lal K. Pandya N. U. Om H. (2022). Antimicrobial and *in-silico* evaluation of novel chalcone and amide-linked 1, 4-disubstituted 1, 2, 3 triazoles. J. Mol. Struct. 1257, 132632. 10.1016/j.molstruc.2022.132632

[B92] Sharma S. P. Vashisht N. Kumar S. Kritika (2018). Ultrasound promoted green synthesis of chalcones of 3-acetylcoumarin. Chem. Sci. Trans. 7, 396–401. 10.7598/cst2018.1502

[B93] Shetye A. P. Pawar M. G. (2017). Microwave assisted synthesis of some new 1, 5-benzodiazepines from chalcones. Pharma Chem. 9, 50–52.

[B94] Shntaif A. H. (2016). Green synthesis of chalcones under microwave irradiation. Int. J. ChemTech Res. 9, 36–39.

[B95] Soozani A. Keivanloo A. Bakherad M. (2017). One-pot synthesis of quinoxaline chalcones from commercially available calcium carbide through palladium-catalyzed coupling reactions. ChemistrySelect 2, 9701–9705. 10.1002/slct.201701803

[B96] Sydnes M. (2014). One-pot reactions: A step towards greener chemistry. Curr. Green Chem. 1, 216–226. 10.2174/2213346101666140221225404

[B97] Tamuli K. J. Sahoo R. K. Bordoloi M. (2020). Biocatalytic green alternative to existing hazardous reaction media: Synthesis of chalcone and flavone derivatives via the Claisen-Schmidt reaction at room temperature. New J. Chem. 44, 20956–20965. 10.1039/d0nj03839c

[B98] Taresh B. (2022). Synthesis, pharmacological activity and uses of chalcone compounds: A review. Sci. J. Med. Res. 6, 43–46. 10.37623/sjomr.v06i21.9

[B99] Thapa P. Upadhyay S. P. Suo W. Z. Singh V. Gurung P. Lee E. S. (2021). Chalcone and its analogs: Therapeutic and diagnostic applications in Alzheimer’s disease. Bioorg. Chem. 108, 104681. 10.1016/j.bioorg.2021.104681 33571811 PMC7928223

[B100] Tran C. T. H. Tran Q. D. Ly D. Nguyen T. T. Nguyen K. X. Nguyen T. T. (2022). A one-pot synthesis of disubstituted thiazoles from chalcone C-H bonds, elemental sulfur, and Glycine ethyl ester. Synlett 33, 555–558. 10.1055/s-0041-1737899

[B101] Tripathi S. Kapoor R. Yadav L. D. S. (2018). Visible light activated radical denitrative benzoylation of β-nitrostyrenes: A photocatalytic approach to chalcones. Adv. Synth. Catal. 360, 1407–1413. 10.1002/adsc.201701559

[B102] Tupare S. D. Pawar R. P. (2020). Synthesis, characterization and biological evaluation of newer chalcones by microwave irradiation. Chem. J. 7, 150–162.

[B103] Villena J. Montenegro I. Said B. Werner E. Flores S. Madrid A. (2021). Ultrasound assisted synthesis and cytotoxicity evaluation of known 2’, 4’-dihydroxychalcone derivatives against cancer cell lines. Food Chem. Toxicol. 148, 111969. 10.1016/j.fct.2021.111969 33421463

[B104] Wei Z. Cue B. W. (2018). Green techniques for organic synthesis and medicinal chemistry. Wiley.

[B105] Weng M.-Y. Xu H. Chen H. Zhang Z. (2021). Liquid-assisted mechanosynthesis of trans-2, 3-dihydropyrroles from chalcones and enaminones. Heterocycles 102, 114–121. 10.3987/com-20-14365

[B106] Wilhelm A. Bonnet S. L. Twigge L. Rarova L. Stenclova T. Visser H. G. (2022). Synthesis, characterization and cytotoxic evaluation of chalcone derivatives. J. Mol. Struct. 1251, 132001. 10.1016/j.molstruc.2021.132001

[B107] Yadav N. Yadav V. B. Ansari M. D. Sagir H. Verma A. Siddiqui I. R. (2019). Catalyst-free synthesis of 2, 3-dihydro-1, 5-benzothiazepines in a renewable and biodegradable reaction medium. New J. Chem. 43, 7011–7014. 10.1039/c8nj05611k

[B108] Yadav P. Lal K. Kumar A. Guru S. K. Jaglan S. Bhushan S. (2017). Green synthesis and anticancer potential of chalcone linked-1, 2, 3-triazoles. Eur. J. Med. Chem. 126, 944–953. 10.1016/j.ejmech.2016.11.030 28011424

[B109] Yadav P. Lal K. Kumar L. Kumar A. Kumar A. Paul A. K. (2018). Synthesis, crystal structure and antimicrobial potential of some fluorinated chalcone-1, 2, 3-triazole conjugates. Eur. J. Med. Chem. 155, 263–274. 10.1016/j.ejmech.2018.05.055 29890388

[B110] Yamamoto T. Yoshimura M. Yamaguchi F. Kouchi T. Tsuji R. Saito M. (2004). Anti-allergic activity of naringenin chalcone from a tomato skin extract. Biosci. Biotechnol. Biochem. 68, 1706–1711. 10.1271/bbb.68.1706 15322354

[B111] Yang Q. Wang R. Han J. Li C. Wang T. Liang Y. (2017). Photo-induced tandem cyclization of 3-iodoflavones with electron rich five-membered heteroarenes. RSC Adv. 7, 43206–43211. 10.1039/c7ra07793a

[B112] Zangade S. Patil P. (2020). A review on solvent-free methods in organic synthesis. Curr. Org. Chem. 23, 2295–2318. 10.2174/1385272823666191016165532

[B113] Żyszka-Haberecht B. Poliwoda A. Lipok J. (2019). ’Structural constraints in cyanobacteria-mediated whole-cell biotransformation of methoxylated and methylated derivatives of 2′-hydroxychalcone. J. Biotechnol. 293, 36–46. 10.1016/j.jbiotec.2019.01.005 30690100

